# Analysis of recreational psychedelic substance use experiences classified by substance

**DOI:** 10.1007/s00213-022-06062-3

**Published:** 2022-01-15

**Authors:** Adrian Hase, Max Erdmann, Verena Limbach, Gregor Hasler

**Affiliations:** 1 Department of Medicine, Faculty of Science and Medicine, University of Fribourg, Fribourg, Switzerland; 2 Faculty of Medicine, University of Rostock, Rostock, Germany; 3 Faculty of Psychology, University of Basel, Basel, Switzerland

**Keywords:** Ayahuasca, DMT, Erowid, Ketamine, LIWC, LSD, MDMA, Psilocybin

## Abstract

**Rationale and objectives:**

Differences among psychedelic substances regarding their subjective experiences are clinically and scientifically interesting. Quantitative linguistic analysis is a powerful tool to examine such differences. This study compared five psychedelic substance report groups and a non-psychedelic report group on quantitative linguistic markers of psychological states and processes derived from recreational use-based online experience reports.

**Methods:**

Using 2947 publicly available online reports, we compared Ayahuasca and N,N-dimethyltryptamine (DMT, analyzed together), ketamine, lysergic acid diethylamide (LSD), 3,4-methylenedioxymethamphetamine (MDMA), psilocybin (mushroom), and antidepressant drug use experiences. We examined word frequencies related to various psychological states and processes and semantic proximity to psychedelic and mystical experience scales.

**Results:**

Linguistic markers of psychological function indicated distinct effect profiles. For example, MDMA experience reports featured an emotionally intensifying profile accompanied by many cognitive process words and dynamic-personal language. In contrast, Ayahuasca and DMT experience reports involved relatively little emotional language, few cognitive process words, increased analytical thinking-associated language, and the most semantic similarity with psychedelic and mystical experience descriptions. LSD, psilocybin mushroom, and ketamine reports showed only small differences on the emotion-, analytical thinking-, psychedelic, and mystical experience-related language outcomes. Antidepressant reports featured more negative emotional and cognitive process-related words, fewer positive emotional and analytical thinking-related words, and were generally not similar to mystical and psychedelic language.

**Conclusion:**

This article addresses an existing research gap regarding the comparison of different psychedelic drugs on linguistic profiles of psychological states, processes, and experiences. The large sample of experience reports involving multiple psychedelic drugs provides valuable information that would otherwise be difficult to obtain. The results could inform experimental research into psychedelic drug effects in healthy populations and clinical trials for psychedelic treatments of psychiatric problems.

**Supplementary Information:**

The online version contains supplementary material available at 10.1007/s00213-022-06062-3.

Psychedelic drugs are commonly associated with recreational, illicit substance use. Consequently, much of the scientific and medical literature has examined psychedelic drugs in the context of substance abuse and its negative consequences. Nonetheless, a small, but growing body of research has recognized the therapeutic potential of psychedelic drugs like Ayahuasca, ketamine, lysergic acid diethylamide (LSD), 3,4-methylenedioxymethamphetamine (MDMA), or psilocybin for psychiatric conditions such as depression and PTSD, especially in patients for whom standard treatments do not work (aan het Rot et al. 2010; Carhart-Harris et al. 2016a; Fuentes et al. 2020; Majumder et al. 2012; Nutt & Carhart-Harris 2020; Palhano-Fontes et al. 2019). As there is no unifying theory of psychedelic drug effects (Swanson 2018), some drugs (e.g., MDMA, ketamine) are not always considered psychedelic. For the purposes of this article, we will treat MDMA and ketamine as psychedelic drugs, consistent with the characterization of psychedelic drugs as having a “…capacity reliably to induce states of altered perception, thought, and feeling that are not experienced otherwise except in dreams or at times of religious exaltation” (Jaffe 1990; Nichols 2016) and being likened to classical psychedelics regarding their fast-acting neuroplastic effects (Olson 2018).

In past studies, psychedelic drugs have often shown promise; for example, a small meta-analysis found that a single intravenous ketamine infusion quickly reduced suicidal ideation in inpatient or emergency care patients with acute suicidality (Bartoli et al. 2017). Similarly, a single dose of psilocybin produced significant and lasting antidepressant effects in patients with treatment-resistant depression (Carhart-Harris et al. 2018a; Carhart-Harris et al. 2016a). Moreover, early research suggested that MDMA may be beneficial in assisting psychotherapy for treatment-resistant PTSD (for a review: Morgan 2020). Other psychological effects of psychedelic drug treatment include increased insights, understanding, self-love, self-worth, and self-compassion; altered self-perception/ego dissolution; feelings of connectedness; transcendental experiences; and emotional spectrum expansion (Breeksema et al. 2020). However, more robust research is needed in the future; as much of this work involved small, highly select samples and/or blinding issues.

Still, due to decades of restrictions and bans on psychedelic drug use (e.g., the 1971 UN Convention on narcotics), there is a relative paucity of research data on the effects of psychedelic drugs on psychological function in nontherapeutic or naturalistic contexts. Furthermore, the rather artificial laboratory or clinical treatment settings of the extant research might have biased effects if one were to try to generalize them to a naturalistic context. Because of this, this study adopted an unobtrusive, naturalistic approach to studying the psychological effects of different psychedelic drugs. Precisely, we used a quantitative semantic-linguistic approach to analyze voluntarily and anonymously published user experience reports for key psychedelic drugs.

As the legal restrictions over the past half-century have not managed to curb recreational drug use (Degenhardt et al. 2008; Reuter & Trautman 2009), there is a myriad of everyday recreational experiences, many of which have been documented in online databases aimed at researching and educating about various kinds of (typically illegal or strictly regulated) drugs. This contrasts a relatively low number of experimental studies on the effects of psychedelic drugs in naturalistic settings, although there are some relevant observational studies (e.g., Bouso et al. 2012; Forstmann et al. 2020). The Internet can be a valuable source of information in this context, as it provides an easy and low-risk option for psychedelic substance users to share their experiences with others. This might result in data to complement standard measures (e.g., questionnaires or clinical interviews), which might in some cases be influenced by reporting bias (e.g., social desirability effects, reluctance to disclose sensitive information) and the rather contrived testing setting of a clinic or laboratory. The latter factor is also relevant in shaping the experience itself, as the effects of psychedelic drugs are essentially context-dependent (Carhart-Harris et al. 2018b). Another strength of analyzing web-based experience reports is that one can compare multiple psychedelic substances at a time (e.g., ketamine, LSD, psilocybin, MDMA), which is rarely achieved in experimental trials. In sum, online reports of recreational psychedelic drug experiences have the potential to provide important supplemental information beyond experimental trials and provide research and therapy considerations. It should be mentioned, though, that online reports may also be biased in some ways, which is why it is important to examine the convergence between results from experimental trials and research relying on online experience reports.

The analysis of experience reports by semantic-linguistic analysis could be done in different ways. One could analyze each report for the appearance of words belonging to predefined categories, some of which are suggestive of psychological states and processes and have been validated in previous research (Pennebaker et al. 2015, 1997). One could also examine the semantic structure of the texts, that is, to analyze what words tend to co-occur in the reports and to calculate the semantic similarity between reports and a reference text based on this information (Deerwester et al. 1990). As these methods allow to quantify written language and transform it into psychologically meaningful variables, they are very suitable for the naturally occurring large amounts of data in experience report databases. They have been used in many contexts (Newman et al. 2008; Pennebaker et al. 2014, 1997), including the study of psychedelic substances (Bedi et al. 2014; Cox et al. 2021; Coyle et al. 2012; Martial et al. 2019; Sapoznikow et al. 2019).

Natural language processing methods have been applied in experimental studies of the effects of psychedelic drugs such as LSD (Sanz et al. 2021), psilocybin (Carrillo et al. 2018), and MDMA (Agurto et al. 2020; Baggott et al. 2015; Bedi et al. 2014; Carrillo et al. 2018; Corey et al. 2016; Marrone et al. 2010; Sanz et al. 2021). Combined with machine learning, they enabled the prediction of the psilocybin treatment response in patients with treatment-resistant depression (Carrillo et al. 2018). The language of post-traumatic stress disorder patients (during therapy) predicted posttreatment symptoms after treatment with MDMA versus placebo, where MDMA produced more treatment-relevant utterances that were negatively related to posttreatment symptom severity (Corey et al. 2016). Moreover, natural language processing-derived topic models predicted long-term abstinence outcomes in individuals who reported having quit alcohol, cannabis, opioid, or stimulant use after a psychedelic substance experience (Cox et al. 2021).

This study sets out to compare different classes of psychedelic substances by means of semantic-linguistic analysis on psychological outcomes extracted from online experience reports aiming to inform other Internet users and contribute to harm reduction. Although previous research has used online experience reports to compare different psychedelic substances, this research did not examine psychological outcomes, instead associating the semantic similarity of the reports with the structural similarity of the respective molecules (Zamberlan et al. 2018). As psychedelic drugs impact emotional and cognitive function (Nichols 2016; Swanson 2018), we analyzed users’ experience reports for the prevalence of different word categories representative of various emotional states and cognitive activity (Pennebaker et al. 2015; Tausczik & Pennebaker 2010). We examined affective words including subcategories for positive emotion, negative emotion, anxiety, and sadness as separate outcomes (Pennebaker et al. 2015). Moreover, we examined words reflective of cognitive processes and analytical thinking (Pennebaker et al. 2015, 1997). We also analyzed experience reports’ semantic similarity to established scales capturing psychedelic or mystical experiences (Hood 1975; Studerus et al. 2010). We thereby aimed to uncover differences and similarities between different psychedelic drugs regarding their psychological effect profiles. To provide a non-psychedelic reference group of experience reports, we also sourced and compared experience reports describing the effects of conventional antidepressant drugs like SSRIs, SNRIs, or tricyclics.

## Method

### Participants and procedure

The sample comprised 2947 psychoactive substance experience reports written by unique report writers. In case a writer had published multiple reports about the substances of interest, their first report was selected for the analysis, discarding additional reports. The writers were 21.2% female, 72.6% male, and 6.2% of unreported gender. Report writers’ mean age was 25.1 years (*SD* = 9.1, 70.3% missing). Age was missing for a majority of the sample because many of the reports were published before the website introduced an age reporting option. The mean weight was *M* = 71.3 kg (*SD* = 16.3 kg, 6.5% missing).

We used the Erowid experience vault (www.erowid.org) for sourcing the reports. This website publishes user-created experience reports for the purposes of education and harm reduction and is curated by staff members who check each report before publication (Erowid & Erowid 2006). In this process, two internal reviewers and one editor read each report and ensure it conforms to the website’s quality standards and guidelines. In this way, incriminating information, incomprehensible content, and advertising or other undesirable content are removed or corrected before publication to the website. Before linguistically analyzing the reports in an automated way, two authors (AH and ME) double-checked and corrected the raw text material for typographic errors unrelated to slang or sociolect and meaningless word repetitions. Another author (VL) then reads 835 reports (150 per psychedelic substance, where available) and, where necessary, deleted any bits of text that did not describe the immediate effects of the substance (the “trip”). We then compared these shortened reports against their full-length versions to ensure the outcomes of interest were not biased by trip-unrelated information in the full reports. In paired-samples *t*-tests, there were no significant differences between the shortened and full reports on any outcome variable (see online supplementary material Table S1), so we analyzed the full-length reports in the main analyses.

We collected experience reports for the following psychedelic drugs: psilocybin-containing mushrooms (*n* = 971 reports), LSD (*n* = 671), MDMA (*n* = 526), N,N-dimethyltryptamine (DMT, *n* = 312), ketamine (*n* = 163), and Ayahuasca (*n* = 68). For a non-psychedelic drug comparison group, we also collected experience reports for conventional antidepressant drugs (*n* = 236). The vast majority of these reports described the acute effects of illicit or off-prescription use of the reported substances, as opposed to describing the effects of long-term ingestion of prescribed dosages. To reduce the number of necessary statistical comparisons and increase statistical power, we analyzed Ayahuasca and DMT together (*n* = 380) due to their considerable overlap (Strassman 2001). Thus, there were six drug groups in the analyses: psilocybin-containing mushrooms (33.0%), LSD (22.8%), MDMA (17.9%), Ayahuasca and DMT (12.9%), conventional antidepressants (8.0%), and ketamine (5.5%). Table 1 provides descriptive information about each drug group.

**Table 1:** Descriptive summary for drugs of interest

Drug group	Self-reported dosage (mg)			Administration route	Administration route *N* (%)
	*M*	*SD*	*N*		
Antidepressants	N/A	N/A	N/A	Oral Insufflation Rectal N/A	201 (85.2%) 23 (9.7%) 1 (0.4%) 11 (4.7%)
Ayahuasca and DMT	49.27	29.20	170	Smoked Oral Vaporization Insufflation IV IM Inhalation Rectal N/A	251 (66.1%) 70 (18.4%) 40 (10.5%) 9 (2.4%) 5 (1.3%) 1 (0.3%) 1 (0.3%) 1 (0.3%) 2 (0.5%)
Ketamine	171.28	157.86	111	Insufflation IM Oral IV Inhalation Subcutaneous Rectal Buccal Smoked N/A	93 (57.1%) 35 (21.5%) 15 (9.2%) 10 (6.1%) 3 (1.8%) 2 (1.2%) 2 (1.2%) 1 (0.6%) 1 (0.6%) 1 (0.6%)
LSD	N/A	N/A	N/A	Oral Sublingual Buccal Insufflation N/A	573 (85.4%) 41 (6.1%) 3 (0.4%) 1 (0.1%) 53 (7.9%)
MDMA	139.50	95.44	71	Oral Insufflation Rectal Inhalation Smoked Sublingual N/A	504 (95.8%) 8 (1.5%) 5 (1.0%) 1 (0.2%) 1 (0.2%) 1 (0.2%) 6 (1.1%)
Psilocybin mushrooms	26.06	36.93	4	Oral Smoked N/A	947 (97.5%) 19 (2.0%) 5 (0.5%)

*N/A* = Not available due to lack of data or non-interpretable data

### LIWC analysis

We imported the downloaded Erowid reports in the text analysis software LIWC2015 (Pennebaker et al. 2015). LIWC2015 analyzes the occurrence of various pre-defined and psychologically meaningful word categories in a body of text. For the analyses in this article, we selected the LIWC categories “affective processes,” “positive emotion,” “negative emotion,” “sadness,” “anxiety,” “cognitive processes,” and “analytical thinking.” We deemed these categories relevant because they present psychological outcomes of interest that might vary between different psychedelic drug experiences; and some of them (anxiety, negative emotion, sadness) have been associated with clinical outcomes in previous research (e.g., depression and anxiety; Eichstaedt et al. 2018; Sonnenschein et al. 2018). Positive emotion and affective words (comprising both positive and negative emotion words) were selected to complement negative emotion words as markers of affective language. The cognitive processes variable (“cause,” “know,” “ought”; Pennebaker et al. 2015) captures cognitive process words related to insight, causation, discrepancy, tentativeness, certainty, and differentiation and has been associated with anxiety (Alvarez-Conrad et al. 2001), physical health (Pennebaker et al. 1997), avoidance of negative experience (Boals & Klein 2016), and gender patterns in language use (Newman et al. 2008). For exploratory purposes, we also summarize the results of the analyses of the remaining LIWC variables in an online supplementary material.

Except for the analytical thinking variable, the LIWC-derived variables reported here are interpreted as the proportion of corresponding category words (e.g., positive emotion words) in the entire text body (i.e., one Erowid report). However, the analytical thinking variable is based on Pennebaker and colleagues’ (2014) categorical-dynamic index, which is based on multiple proportions of different word categories. For this variable, LIWC divides its eight function word categories into two groups (articles and prepositions versus personal pronouns, impersonal pronouns, auxiliary verbs, conjunctions, adverbs, and negations) and then subtracts the summed proportions for the second group from the summed proportions for the first group (plus a constant), making the interpretation more abstract. The analytical thinking variable has been found to correlate positively with college grades and is thought to reflect heightened abstract thinking (via greater article use) and cognitive complexity on the positive end (via greater preposition use; Pennebaker et al. 2014). On the negative end, the creators interpreted the variable as representing a dynamic or narrative language style. This might be relevant in the current study because psychedelics are thought to impair the evaluative dimension of abstract thought (Bayne & Carter 2018). The previously mentioned LIWC variables were thus chosen as continuous outcome measures to compare substances on.

### Latent semantic analysis

Next to the word frequency-based LIWC analyses, we also analyzed the reports with latent semantic analysis (LSA), a natural language processing technique that quantifies the semantic similarity between two given text corpora. We examined the semantic similarity between the Erowid reports and two reference texts to operationalize the similarity between report authors’ experiences and the experiences that the reference texts were designed to capture. Precisely, the reference texts were scales for measuring psychedelic and mystical experiences, respectively: the OAV scale (Studerus et al. 2010) and Hood’s Mysticism scale (Hood 1975). We ran LSA in Python 3.8 (using Spyder 5.0.5). First, we preprocessed the text materials with the *nltk* part-of-speech tagger and lemmatized the text corpus using the *WordNet* lemmatizer contained in the *nltk* library (Bird et al. 2009). We then ran two LSAs using the *gensim* library (Řehůřek & Sojka 2010) on the Erowid report corpus to determine semantic similarity values for each Erowid report with reference to the OAV scale (for psychedelic experiences) and Hood’s M scale (for mystical experiences), respectively. This allowed for group-level comparisons of semantic similarity between report groups, akin to previous research into the effects of psychedelic substances (Bedi et al. 2014; Martial et al. 2019).

### Statistical analyses

To examine the association between substance and word frequency, we conducted ANOVAs with each of the selected outcomes as dependent variable (continuous; LIWC word category frequencies and LSA similarity index values) and substance as the independent variable (categorical, i.e., antidepressants vs Ayahuasca and DMT vs ketamine vs MDMA vs LSD vs psilocybin mushrooms). Significant effects were followed up with Bonferroni-corrected post hoc tests. The ANOVA assumptions were checked by means of visual inspection of histograms and Q-Q plots (normality assumption) and Levene’s test (homoscedasticity assumption). In case of violations, we used appropriate robust alternatives (heteroscedastic one-way ANOVA for trimmed means in case of heteroscedasticity; Kruskal–Wallis test in case of non-normality or both assumptions being violated) and their corresponding post hoc tests (Yuen’s test for trimmed-means and Dunn test for nonparametric comparisons). We also analyzed demographic variables in this way, although we used a chi-squared test for the analysis of gender by report group (with *z*-tests for independent proportions as potential follow-up comparisons). We used RStudio (version 1.3.1073) for all analyses, and the *ggstatsplot* package in R for data visualization (Patil 2018; R Core Team 2020). The significance level was *α* = 0.05.

## Results

Outcome variable assumption checks revealed problems with the normality assumption on negative emotion, anxiety, and sadness words and problems with homoscedasticity on positive emotion, negative emotion, anxiety, sadness words, OAV scale semantic similarity, and Hood’s M scale semantic similarity scores. As a consequence, we used *ggstatsplot*’s robust option for positive emotion words, OAV scale similarity index, and Hood’s M scale similarity index and the nonparametric option for negative emotion, anxiety, and sadness words and interpreted the results appropriately. Due to normality and homoscedasticity violations on age and weight, respectively, we also used appropriate alternative tests for these variables.

Table 2 compares report writers’ demographic data by substance, and online supplementary Figs. S1 and S2 visualize significant differences between drugs on age and weight. There were significant effects for the distribution of age in years, *χ*^*2*^ (5) = 48.86, *p* < 0.001 and *ε*^2^_ordinal_ = 0.06; weight in kilograms, *F*_trimmed-means_ (5,475.72) = 8.83, *p* < 0.001, *ξ*^2^ = 0.18; and gender, *χ*^*2*^ (5) = 118.65, *p* < 0.001. LSD report writers were of the youngest age (Median = 20.00), being significantly younger than Ayahuasca and DMT (Median = 24.00, *p* < 0.001), psilocybin (Median = 23.00, *p* < 0.001), and antidepressant report authors (Median = 24.00, *p* = 0.001). MDMA report writers (Median = 21.00) were significantly younger than Ayahuasca and DMT (*p* < 0.001), psilocybin (*p* = 0.007), and antidepressant report authors (*p* = 0.008). MDMA report authors had the lowest trimmed-mean weight (*M* = 66.26), being significantly lighter than Ayahuasca and DMT (*M* = 72.56, *p* < 0.001), psilocybin mushroom (*M* = 69.38, *p* = 0.045), and antidepressant report authors (*M* = 71.73, *p* = 0.006). Ayahuasca and DMT report authors were also significantly heavier than LSD report authors (*M* = 68.56, *p* = 0.007). Based on the expected cell proportions for gender, there were significantly fewer female report writers than expected in the Ayahuasca and DMT (*p* < 0.001), ketamine (*p* < 0.01), and psilocybin mushroom report groups (*p* < 0.001). In the MDMA group, there were significantly more female report writers than expected (*p* < 0.001).

**Table 2:** Summary of sample demographics

	Age (years)			Weight (kg)			Gender	
Drug	*M*	*SD*	*N*	*M*	*SD*	*N*	*N*_Male_ (%)	*N*_Female_ (%)
Antidepressants	27.95	10.42	57	74.63	18.74	229	169 (74.45%)	58 (25.55%)
Ayahuasca and DMT	26.09	8.11	226	74.01	15.75	357	310 (84.93%)**	55 (15.07%)
Ketamine	24.69	10.36	61	71.85	16.34	148	133 (86.36%)*	21 (13.64%)
LSD	22.45	6.77	186	70.22	16.12	623	482 (78.50%)	132 (21.50%)
MDMA	23.06	7.82	123	69.69	18.78	467	295 (61.08%)	188 (38.92%)**
Psilocybin mushrooms	26.88	10.83	221	70.99	14.47	932	750 (81.34%)**	172 (18.66%)
Overall	25.11	9.10	817	71.33	16.34	2756	2139 (77.36%)	626 (22.64%)
	*χ*^*2*^ (5) = 48.86, *p* < .001, *ε*^2^_ordinal_ = .06, *CI* 95% (.03, .09)			*F*_trimmed-means_ (5475.72) = 8.83, *p* < .001, *ξ*^2^ = .18, *CI* 95% (.10, 0.26)			*χ*^*2*^ (5) = 102.11, *p* < .001	

^*^Based on the expected cell proportions, this gender is overrepresented in this report group, *p* < .01; **Based on the expected cell proportions, this gender is overrepresented in this report group, *p* < .001

Figure S3 displays the trimmed-means ANOVA results and significant pairwise comparisons for the affective process words outcome. There was a significant report group effect, *F*_trimmed-means_ (5503.16) = 75.66, *p* < 0.001 and *ξ*^2^ = 0.45, *CI* 95% (0.40, 0.51). Bonferroni-corrected post hoc tests showed that MDMA reports were associated with the highest proportion of affective process words (*M* = 5.26), significantly higher than in psilocybin mushroom (*M* = 4.28, *p* < 0.001), LSD (*M* = 4.26, *p* < 0.001), Ayahuasca and DMT (*M* = 3.71, *p* < 0.001), and ketamine reports (*M* = 3.71, *p* < 0.001). Moreover, antidepressant reports featured a significantly higher proportion of affective process words than Ayahuasca and DMT (*p* < 0.001), ketamine (*p* < 0.001), LSD (*p* < 0.001), and psilocybin mushroom reports (*p* < 0.001). Finally, Ayahuasca and DMT and ketamine reports featured a significantly lower proportion of affective process words than LSD and psilocybin mushroom reports, respectively (all *p* < 0.001).

**Fig. 1:**
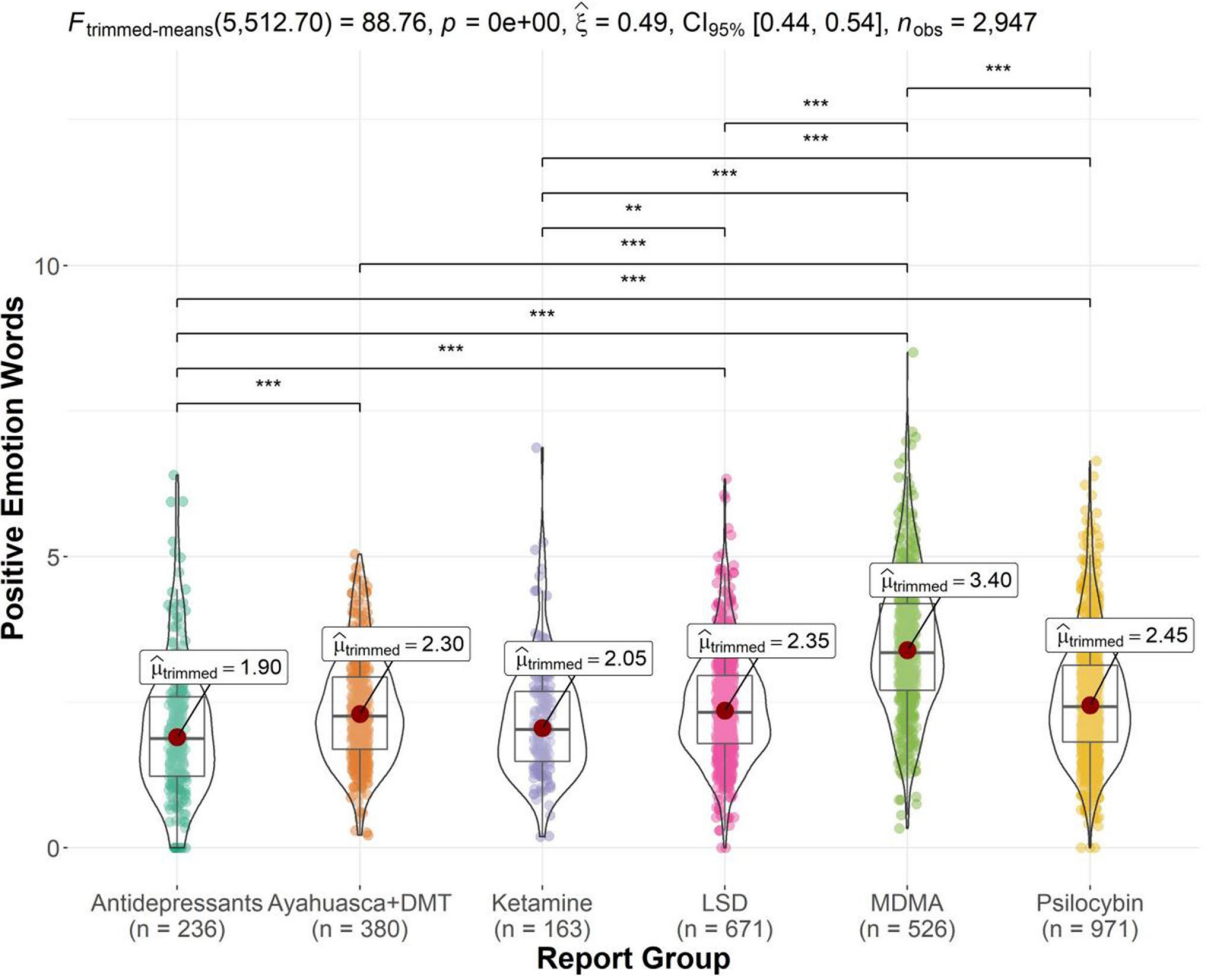
Violin plot of positive emotion word proportion by report group (significant comparisons denoted by **p < .01, ***p < .001). MDMA reports featured the highest proportion of positive emotion words, significantly higher than that of every other substance. Antidepressant reports featured the lowest proportion of positive emotion words, significantly lower than all other groups except ketamine reports. Ketamine reports featured a significantly lower proportion than psilocybin mushroom and LSD reports

Figure 1 displays trimmed-means ANOVA results and significant pairwise comparisons for the positive emotion words outcome. There was a significant report group effect, *F*_trimmed-means_ (5512.70) = 88.76, *p* < 0.001 and *ξ*^2^ = 0.50, *CI* 95% (0.43, 0.57). Post hoc comparisons showed that the MDMA reports (*M* = 3.40) featured a significantly higher positive emotion word proportion than the psilocybin mushroom (*M* = 2.45, *p* < 0.001), LSD (*M* = 2.35, *p* < 0.001), Ayahuasca and DMT (*M* = 2.30, *p* < 0.001), ketamine (*M* = 2.05, *p* < 0.001), and antidepressant reports (*M* = 1.90, *p* < 0.001). Furthermore, the antidepressant reports featured a significantly lower positive emotion word proportion than Ayahuasca and DMT (*p* = 0.001), LSD (*p* < 0.001), and psilocybin mushroom reports (*p* < 0.001). Finally, ketamine reports featured significantly a lower positive emotion word proportion than psilocybin mushroom (*p* < 0.001) and LSD reports (*p* = 0.027).

**Fig. 2:**
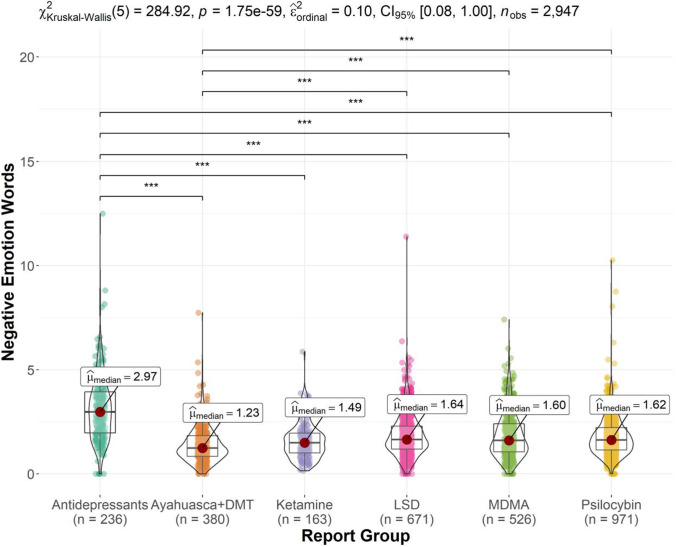
Violin plot of negative emotion word proportion by report group (significant comparisons denoted by ***p < .001). Antidepressant reports featured significantly higher proportions of negative emotion words than the other report groups. Ayahuasca and DMT reports featured significantly lower proportions than psilocybin mushroom, MDMA, and LSD reports

Figure 2 displays the Kruskal–Wallis test results and significant pairwise comparisons for the negative emotion words outcome. There was a significant report group effect, *χ*^*2*^ (5) = 284.92, *p* < 0.001 and *ε*^2^_ordinal_ = 0.10, *CI* 95% (0.08, 0.12). Post hoc comparisons showed that Ayahuasca and DMT reports (*Median* = 1.23) featured a significantly lower median proportion of negative emotion words than MDMA (*M* = 1.60, *p* < 0.001), psilocybin mushroom (*Median* = 1.62, *p* < 0.001), LSD (*M* = 1.64, *p* < 0.001), and antidepressant reports (*Median* = 2.97, *p* < 0.001). Antidepressant reports (*Median* = 2.97) featured a higher median proportion of negative emotion words than ketamine reports (*Median* = 1.49, *p* < 0.001) and all other report groups (all *p* < 0.001).

Figure S4 displays the Kruskal–Wallis test results and significant pairwise comparisons for the sadness words outcome. There was a significant report group effect, *χ*^*2*^ (5) = 79.44, *p* < 0.001 and *ε*^2^_ordinal_ = 0.03, *CI* 95% (0.02, 0.04). Post hoc comparisons showed that Ayahuasca and DMT reports (*Median* = 0.23) featured a lower median proportion of sadness words than LSD (*Median* = 0.26, *p* = 0.029), psilocybin mushroom (*Median* = 0.28, *p* = 0.001), MDMA (*Median* = 0.30, *p* = 0.001), and antidepressant reports (*Median* = 0.57, *p* < 0.001). Antidepressant reports featured a higher median proportion of sadness words than each of the other report groups (all *p* < 0.001).

Figure S5 displays the Kruskal–Wallis test results and significant pairwise comparisons for the anxiety words outcome. There was a significant report group effect, *χ*^*2*^ (5) = 21.70, *p* = 0.001 and *ε*^2^_ordinal_ = 0.01, *CI* 95% (0.00, 0.02). Post hoc comparisons showed that ketamine reports (*Median* = 0.27) featured a lower median proportion of anxiety words than psilocybin mushroom (*Median* = 0.42, *p* = 0.002), LSD (*Median* = 0.41, *p* = 0.010), and antidepressant reports (*Median* = 0.46, *p* = 0.006).

**Fig. 3:**
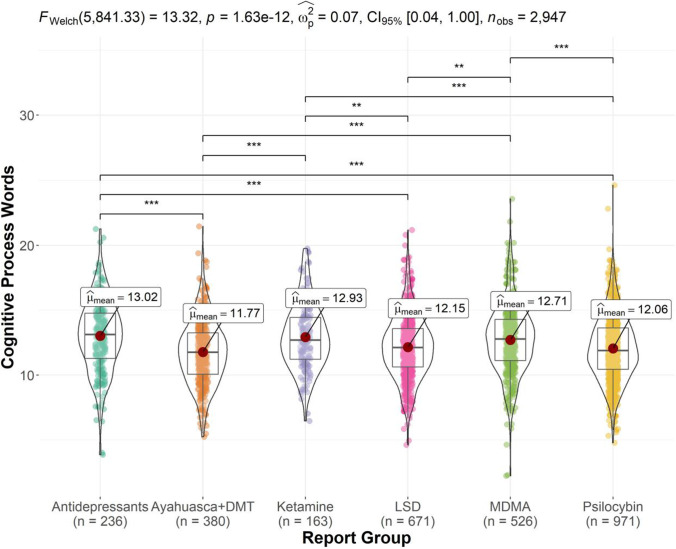
Violin plot of cognitive process word proportion by report group (significant comparisons denoted by **p < .01, ***p < .001). Ayahuasca and DMT reports featured the lowest mean proportion of cognitive process words, followed by psilocybin mushroom and LSD reports. The lowest three proportions (Ayahuasca and DMT, psilocybin mushroom, and LSD reports) were each significantly lower than those of antidepressant, ketamine, and MDMA reports

Figure 3 displays the ANOVA results and significant pairwise comparisons for the cognitive process words outcome. There was a significant report group effect, *F* (5,2941) = 14.29, *p* < 0.001 and *η*^*2*^_p_ = 0.02, *CI* 95% (0.01, 0.03). Bonferroni-corrected post hoc tests showed that Ayahuasca and DMT reports were associated with the lowest proportion of cognitive process words (*M* = 11.77), significantly lower than that of MDMA (*M* = 12.71, *p* < 0.001), ketamine (*M* = 12.93, *p* < 0.001), and antidepressant reports (*M* = 13.02, *p* < 0.001). Psilocybin mushroom reports (*M* = 12.06) featured a significantly lower proportion of cognitive process words than MDMA (*p* < 0.001), ketamine (*p* = 0.001), and antidepressant reports (*p* < 0.001). LSD reports (*M* = 12.15) featured a significantly lower proportion of cognitive process words than MDMA (*p* = 0.002), ketamine (*p* = 0.006), and antidepressant reports (*p* < 0.001).

**Fig. 4:**
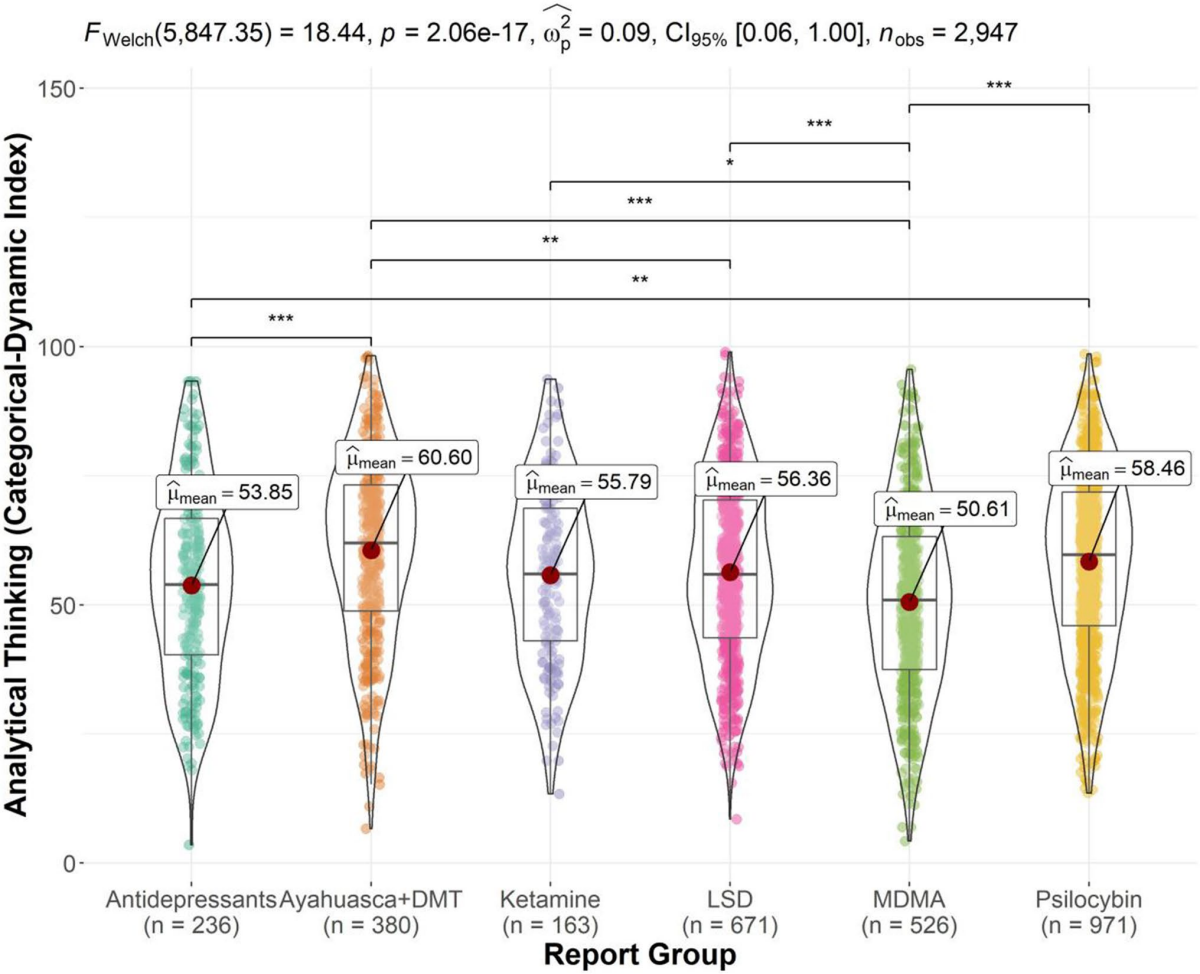
Violin plot of analytical thinking index scores by report group (significant comparisons denoted by *p < .05, **p < .01, ***p < .001). MDMA reports featured the lowest mean analytical thinking index score; significantly lower than that of Ayahuasca and DMT, psilocybin mushroom, LSD, and ketamine reports. Antidepressant reports featured significantly lower scores than Ayahuasca and DMT and psilocybin mushroom reports. LSD reports featured a significantly lower analytical thinking index mean score than Ayahuasca and DMT reports

Figure 4 displays the ANOVA results and significant pairwise comparisons for the analytical thinking index. There was a significant substance group effect,^1^*F* (5, 2941) = 19.31, *p* < 0.001, *η*^2^_p_ = 0.03, and *CI* 95% (0.02, 0.04). Bonferroni-corrected post hoc tests showed that Ayahuasca and DMT reports scored highest on the analytical thinking index (*M* = 60.60), significantly higher than LSD (*M* = 56.36, *p* = 0.003), antidepressant (*M* = 53.85, *p* < 0.001), and MDMA reports (*M* = 50.61, *p* < 0.001). MDMA reports scored significantly lower than ketamine (*M* = 55.79, *p* = 0.016), LSD (*p* < 0.001), and psilocybin mushroom reports (*M* = 58.46, *p* < 0.001). Psilocybin mushroom reports also scored significantly higher than antidepressant reports (*p* = 0.005).

**Fig. 5:**
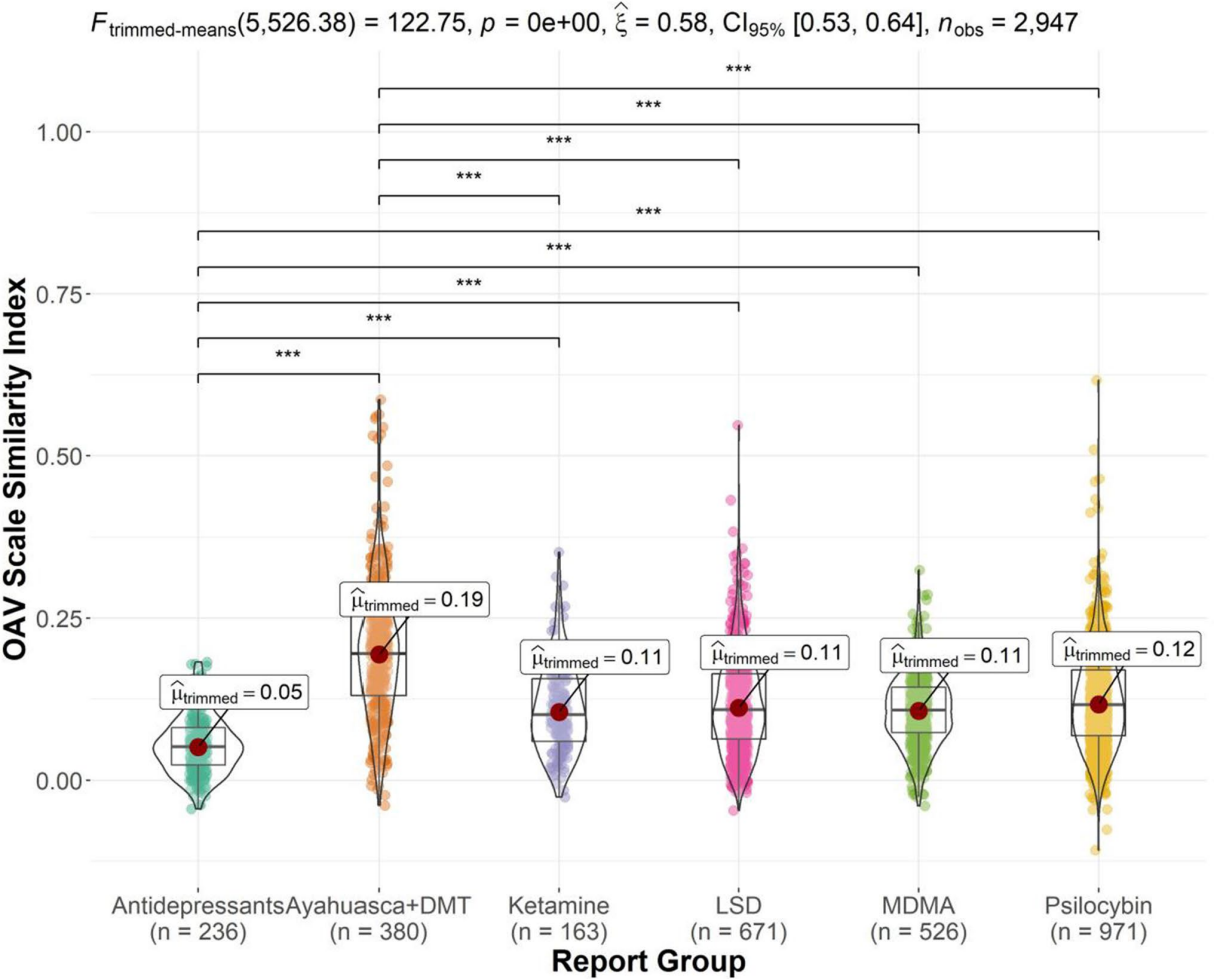
Violin plot of OAV scale latent semantic analysis similarity index scores by report group (significant comparisons denoted by ***p < .001). Ayahuasca and DMT reports featured significantly more semantic similarity to the OAV scale than each of the remaining report groups. Antidepressant reports featured significantly less semantic similarity to the OAV scale than each of the remaining report groups

Figure 5 displays the trimmed-means ANOVA results and significant pairwise comparisons for the OAV scale similarity index. There was a significant report group effect, *F* (5526.38) = 122.75, *p* < 0.001, *ξ*^2^ = 0.58, and *CI* 95% (0.51, 0.65). Yuen’s trimmed-means tests showed that Ayahuasca and DMT reports featured the greatest semantic similarity with the OAV scale (*M* = 0.19), significantly greater than antidepressant (*M* = 0.05, *p* < 0.001), ketamine (*M* = 0.11, *p* < 0.001), LSD (*M* = 0.11, *p* < 0.001), MDMA (*M* = 0.11, *p* < 0.001), and psilocybin mushroom reports (*M* = 0.12, *p* < 0.001). Antidepressant reports featured the least semantic similarity with the OAV scale, significantly less than ketamine (*p* < 0.001), LSD (*p* < 0.001), MDMA (*p* < 0.001), and psilocybin mushroom reports (*p* < 0.001).

**Fig. 6:**
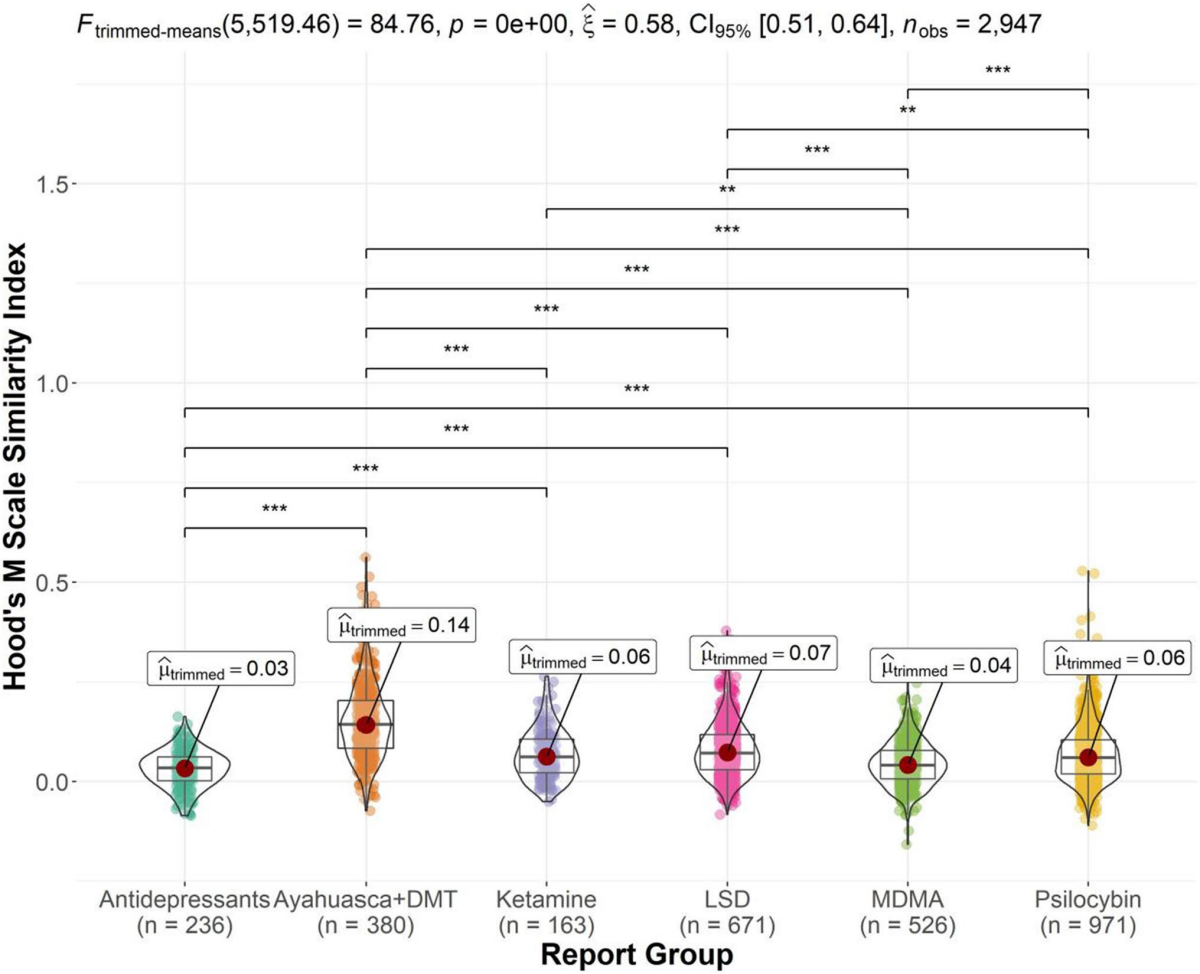
Violin plot of Hood’s M scale latent semantic analysis similarity index scores by report group (significant comparisons denoted by **p < .01, ***p < .001). Ayahuasca and DMT reports featured significantly more semantic similarity to Hood’s M scale than each of the remaining report groups. LSD reports featured significantly more semantic similarity than antidepressant, MDMA, and psilocybin mushroom reports. MDMA reports featured significantly less semantic similarity than ketamine and psilocybin mushroom reports. Antidepressant reports featured significantly less semantic similarity to Hood’s M scale than ketamine and psilocybin mushroom reports

Figure 6 displays the trimmed-means ANOVA results and significant pairwise comparisons for the Hood’s M scale similarity index. There was a significant substance group effect, *F* (5519.46) = 84.76, *p* < 0.001 and *ξ*^2^ = 0.57, *CI* 95% (0.50, 0.65). Yuen’s trimmed-means tests showed that Ayahuasca and DMT reports featured the greatest semantic similarity with Hood’s M scale (*M* = 0.14), significantly greater than antidepressant (*M* = 0.03, *p* < 0.001), ketamine (*M* = 0.06, *p* < 0.001), LSD (*M* = 0.07, *p* < 0.001), MDMA (*M* = 0.04, *p* < 0.001), and psilocybin mushroom reports (*M* = 0.06, *p* < 0.001). Antidepressant reports featured the least semantic similarity with Hood’s M scale, significantly less than ketamine (*p* < 0.001), LSD (*p* < 0.001), and psilocybin mushroom reports (*p* < 0.001). Also, MDMA reports scored lower on similarity with Hood’s M scale than ketamine (*p* = 0.031), LSD (*p* < 0.001) and psilocybin reports (*p* < 0.001); and psilocybin reports scored lower than LSD reports (*p* = 0.038).

## Discussion

The present study analyzed anonymous online experience reports linked to the use of various psychedelic drugs. The exploratory analyses revealed clear differences between report groups, which amounted to very small (anxiety words), small (sadness words), medium (affective process, negative emotion, positive emotion, and cognitive process words; analytical thinking index), and large (OAV scale and Hood’s M scale semantic similarity) effect sizes (Field 2013; Wilcox & Tian 2011). These effect sizes should be kept in mind as the different language profiles of the different report groups will be discussed step-by-step below.

Ayahuasca and DMT reports contained relatively little affective language, notably on negative emotion and sadness words. They also featured relatively few cognitive process words and high scores on the analytical thinking variable. Given that some aspects of the Ayahuasca experience (nausea, vomiting, diarrhea, bad taste of the beverage; Hamill et al. 2019) would be expected to feel quite unpleasant, it is somewhat surprising that this was not reflected in the reports via a greater proportion of negative emotional language. This counterintuitive finding could be explained with Ayahuasca reports making up less than a fifth of the reports in the Ayahuasca and DMT group. Indeed, when we analyzed the proportion of negative emotion words for Ayahuasca and DMT reports separately, we found that DMT reports featured significantly a lower median proportion of negative emotion words than the other report groups, whereas Ayahuasca reports featured a higher median proportion of negative emotion words (albeit not significantly different from the other psychedelic report groups; see Fig. S6). Hence, despite being a key component of the psychedelic effects of Ayahuasca, DMT may not be responsible for Ayahuasca’s negative side effects. Ayahuasca and DMT reports also featured the greatest semantic similarity with the referential psychedelic and mystical experience scales. This might indicate that on average, Ayahuasca and DMT reports described the most psychedelic and mystical experiences. The latter would be consistent with the findings of Griffiths et al. (2019), who found that complete mystical experiences were most frequent, and mystical experience questionnaire scores the highest in a group of users who reported having had a God encounter after consuming DMT, relative to having consumed psilocybin, LSD, or no drug (Fig. S7 shows that the finding remains consistent even when examining DMT reports separately).

Ketamine reports were associated with relatively little affective language, particularly on positive emotion and anxiety words. As controlled research has observed facial affective flattening on ketamine (Pomarol-Clotet et al. 2006), one could speculate whether the language and facial expressions associated with ketamine’s effects both reflect an underlying emotional blunting. Although one review lists emotional blunting as a potential side effect of ketamine (Caddy et al. 2015), more research is needed to test this idea. Ketamine reports also featured relatively many cognitive process words. This might be because report writers noticed changes in their cognitive function, which they then discussed in their reports. The direction of these changes could be positive or negative. For example, affective flattening could lead to less interference of negative emotion with cognitive processes (Scheidegger et al. 2016), thereby increasing cognitive processing and reports thereof. Contrarily, it might also be that ketamine produced cognitive impairments (Morgan et al. 2004), and this in turn led to the increased cognitive process words observed in the current study. As could generally be the case for this study’s results, an alternative explanation for the cognitive process words finding could be that the difference was not due to the drug’s effects themselves but rather due to some unknown third factor like selection bias. For example, individuals who choose to use ketamine might habitually use less cognitive process words than individuals who choose to use other drugs. Given that ketamine dosages in these reports were likely greater than the low-dose ketamine and esketamine therapeutic use in current clinical practice (e.g., Bartoli et al. 2017), these results may not apply to clinical settings.

LSD reports featured the highest proportion of negative emotion words among the psychedelic drug report groups, although only the difference with Ayahuasca and DMT reports reached statistical significance, and the proportion was nearly half that of antidepressant reports. The same pattern was observed on sadness words and on anxiety words; LSD reports were significantly different from ketamine only. LSD reports were on neither of the extreme ends of the cognitive processes and analytical thinking variables. LSD reports were associated with fewer cognitive process words than ketamine MDMA, and antidepressant reports, lower analytical thinking scores than Ayahuasca and DMT reports, and higher analytical thinking scores than MDMA reports. The finding of a high negative emotion word proportion in LSD reports might have been due to writers’ direct experience of negative emotions, or an analytical focus on their own, or others’ negative emotions in other situations. The latter explanation would be consistent with experimental findings of enhanced emotional empathy under the influence of LSD (Dolder et al. 2016). Interestingly, Dolder and colleagues also found that LSD reduced the recognition of negative emotion, which would support the idea that the observed effects in this study may have been due to a conceptual-analytical focus on, rather than an increased experience of, negative emotion.

MDMA reports were characterized by relatively affective language. This was consistent with other natural language processing-based research (Bedi et al. 2014), and in part due to a high proportion of positive emotion words, consistent with findings from previous LIWC-based experimental research (Bedi et al. 2014; Wardle & de Wit 2014). MDMA reports also featured a relatively high proportion of negative emotion words, which is inconsistent with the results reported by Wardle and de Wit (2014), who found no differences between MDMA and placebo on negative emotion word frequency. In other research, there were mixed results as MDMA was associated not only with increased anxiety but also with decreased recognition of specific negative emotions (sadness and anger) in photos of human faces (Kirkpatrick et al. 2014). There also were a relatively high proportion of cognitive process words and the lowest score on the analytical thinking variable. The cognitive process words finding is interesting, because previous research has demonstrated cognitive decrements due to MDMA (Parrott & Lasky 1998). Thus, it could be that writers were aware of their cognitive difficulties, and writing about this in the reports increased the frequency of cognitive process words. The analytical thinking variable finding indicates that MDMA reports were associated with language rich in personal pronouns, impersonal pronouns, auxiliary verbs, conjunctions, adverbs, and negations and low in articles and prepositions (Pennebaker et al. 2014). This represents a more dynamic and personal, as opposed to analytical and impersonal narration style; potentially due to intensified emotional states on MDMA. Lastly, of all the psychedelic substance report groups, MDMA reports featured the least semantic similarity with Hood’s mystical experience scale (comparable to antidepressant reports), which seems consistent with previous research results (Lyvers & Meester 2012).

Psilocybin mushroom reports were not on the extreme ends of any outcome variable. Notably, they featured less affective language than MDMA (also on positive emotion words) and antidepressant reports (due to negative emotion and sadness words, as the reverse was true for positive emotion words) but more than Ayahuasca and DMT (also on negative emotion and sadness words) and ketamine reports (also on positive emotion and anxiety words). There also was a lower proportion of cognitive process words than in MDMA, ketamine, and antidepressant reports and a higher analytical thinking score than in MDMA and antidepressant reports. There was more semantic similarity to Hood’s mysticism scale relative to MDMA and antidepressant reports but less similarity relative to Ayahuasca and DMT and LSD reports, as well as less similarity to the OAV scale relative to Ayahuasca and DMT reports. One could compare these results to those of a survey study of individuals who had a God encounter (Griffiths et al. 2019), which found that psilocybin-induced God encounters reportedly featured less strong and less frequent complete mystical experiences than DMT-induced encounters (whereas there was no difference to LSD and Ayahuasca experiences; Griffiths et al. 2019).

Antidepressant reports, which were included as a non-psychedelic substance comparison regarding Erowid users’ language, featured a distinct profile. They featured a relatively high proportion of affective language (only outscored by MDMA reports), but in contrast to MDMA reports, this was due to a significantly higher proportion of negative emotion and sadness words relative to the other report groups, the proportion of positive emotion words being lower than that in the other reports. This finding might be naturally explained by antidepressant users’ higher prevalence of depression and associated negative emotional biases. Antidepressant reports also featured the highest proportion of cognitive process words, which one could attribute to cognitive characteristics of depression like increased rumination. On analytical thinking, antidepressant reports featured lower scores than Ayahuasca and DMT and psilocybin mushroom reports, but did not differ from the remaining psychedelic drugs (and scored higher than MDMA reports), indicating a notable difference between psychedelic substances on this dimension. Antidepressant reports also featured less semantic similarity with the psychedelic and mystical experience scales than the psychedelic drug report groups.

Though no prior study has compared the present combination of psychedelic substances on psycholinguistic variables like this study did, previous research used similar outcomes or analytic methods. Studies that examined the effects of psychedelic drugs on the psychological states or processes underlying the psycholinguistic outcomes of this study reported emotional effects of psychedelic drugs, such as an emotionally intensifying profile on MDMA (Agurto et al. 2020; Bedi et al. 2014), decreased negative emotions like sadness on ketamine (Hasler et al. 2020), increased 5D-ASC blissful state and increased emotional excitability with a bias toward positive emotion on LSD (Carhart-Harris et al. 2016a; Liechti et al. 2017; Schmid et al. 2015), and emotional excitability on psilocybin (Hasler et al. 2004). Furthermore, some research also observed cognitive disorganization on LSD (Carhart-Harris et al. 2016b). However, few studies compared psychedelic drugs against each other. Holze et al. (2020) and Liechti et al. (2017) compared LSD against MDMA on the Mystical Experience Questionnaire and found a seemingly contradictory result to the present study, where LSD was associated with higher “deeply felt positive mood” and “positive mood” subscale scores than MDMA. Furthermore, LSD was associated with higher 5D-ASC blissful state scores (Holze et al. 2020). Zamberlan et al. (2018) compared many different psychedelic drugs with latent semantic analysis of Erowid experience reports but only correlated the semantic similarity (to other report groups) with the molecular structural similarity between drugs to infer something about the effects that molecular differences might have on psycholinguistic outcomes. These results emphasize that ideally, experimental research should compare different psychedelic drugs directly on outcomes derived from both natural language processing methods and validated questionnaires to (a) determine differences between psychedelic drugs and (b) the relationship between linguistic indicators of psychological variables and self-report measures of those same variables.

This study was limited in several ways. First, the design and analyses of the study did not permit causal inference. The fact that there was no control over the administration, dosage, and quality of psychedelic substances, nor over the time at which the experience was recalled and reported, shows that these results should only be interpreted as indirect indicators of potential substance group effects. For example, it is not clear whether the use of the examined substances produced the differences in language that the analyses detected or whether this was due to a third variable predisposing individuals toward recreational use of a specific substance that also influenced the linguistic outcomes. We did, however, check demographic variables (age, weight, and gender) and found significant differences between substances. For example, Ayahuasca and DMT, psilocybin, and antidepressant reports were associated with older age than LSD and MDMA reports. Ayahuasca and DMT reports were associated with heavier report authors than LSD and MDMA reports, the latter also involving significantly lighter authors than psilocybin and antidepressant reports. Females were underrepresented in Ayahuasca and DMT, ketamine, and psilocybin reports and overrepresented in MDMA reports. Some of these effects might be interrelated, and some of them might also be related to the findings on the linguistic outcomes. For example, the lower weight of MDMA report writers might be due to the relative overrepresentation of women in this group, which might in turn explain part of the more emotional language in this group (Newman et al. 2008). Rigid experimental research balancing the proportions of men and women per substance could provide insights into these relationships, although such research should be very difficult to conduct with the combination of substances examined here. Future research could also monitor other potential sources of bias, such as cultural background or socioeconomic status. Another limitation concerns the antidepressant report group, which is not an ideal control condition, as would be a placebo group in a hypothetical controlled experimental version of this study. Given the constraints of the study design, though, the antidepressant reports did provide useful information about the language of Erowid authors who did not use psychedelic substances.

Given the absence of experimental data regarding the psycholinguistic effects of different psychedelic drugs, this study provided valuable insights. In light of its findings and limitations, future research could build on this study by conducting a methodologically robust experimental trial examining the substances reviewed here, and a potential control or placebo substance, using free speech transcripts or written texts produced during the experience. Another limitation concerns the dearth of demographic information about the sample, as more data could help explain who uses psychedelic drugs and with what motivation. The available descriptive information (i.e., age, gender, weight) provided some insight (e.g., women, lighter, and younger people being relatively overrepresented in MDMA reports; Ayahuasca and DMT report writers being relatively old; Ayahuasca and DMT, ketamine, and psilocybin report groups being relatively male), but more variables might be needed to show that differences between substances persist when controlling for individual differences. Future research could thus examine demographic variables not available here (e.g., educational/social status, cultural background, substance preference).

## Conclusion

This study examined differences in linguistic markers of psychological function between different psychedelic drug experience reports. These differences indicated certain psychological effect profiles, although more controlled studies are necessary to more closely examine these profiles and replicate the current results. The study indicates that some drugs may have an emotionally intensifying profile accompanied by many cognitive process words and dynamic, personal language (MDMA), whereas others are characterized by reductions of emotional language, relatively few cognitive process words, and increases in analytical thinking-associated language (Ayahuasca and DMT), and again others show a mixed effect profile (ketamine, LSD, psilocybin mushrooms). With this article, we hope to contribute to the development of psychedelic-assisted psychotherapy or psychiatric treatments for psychiatric problems, for example, by optimizing the selection of appropriate substances based on patient needs.

## Supplementary Information

Below is the link to the electronic supplementary material.

Supplementary file1 (DOCX 3771 KB)

## Footnotes

1 This effect did not survive the adjustment for age, weight, gender, and their interactions with report group in an ANCOVA to examine the effects of these control variables. The same was the case for the report group effect on anxiety words, although this interpretation would be questionable given the assumption violations on anxiety words. The effects on the other outcomes survived the adjustment for the abovementioned covariates.
