# Supplementary material for: Analysis of recreational psychedelic substance use experiences classified by substance
Source: Psychopharmacology (Berl). 2022 Jan 15;239(2):643–59. doi: 10.1007/s00213-022-06062-3 (PMC8799548; doi:10.1007/s00213-022-06062-3)
Supplement: Supplementary file 1 — Supplementary file1 (DOCX 3771 KB) [file 213_2022_6062_MOESM1_ESM.docx]

**Supplementary Online Material**

Analysis of Recreational Psychedelic Substance Use Experiences Classified by Substance

For Publication in *Psychopharmacology*

Adrian Hase^1^

Max Erdmann^2^

Verena Limbach^3^

Gregor Hasler^1^

^1^ Department of Medicine, Faculty of Science and Medicine, University of Fribourg, Fribourg, Switzerland

^2^ Faculty of Medicine, University of Rostock, Rostock, Germany

^3^ Department of Psychology, Faculty of Humanities, University of Basel, Basel, Switzerland

Corresponding author: Adrian Hase, e-mail: adrian.hase@unifr.ch

**Table S1**

*Paired T-Tests Comparing Shortened and Full Reports on LIWC Outcome Variables*

|  | Complete Reports | | Shortened Reports | |  |  |  | 95% *CI* | |
| --- | --- | --- | --- | --- | --- | --- | --- | --- | --- |
| Outcome | *M* | *SD* | *M* | *SD* | *t* | *p* | *d* | *LB* | *UB* |
| Affective Processes | 4.34 | 1.52 | 4.34 | 1.55 | -0.80 | .42 | -0.03 | -0.10 | 0.04 |
| Positive Emotion | 2.59 | 1.09 | 2.58 | 1.12 | 1.00 | .32 | 0.03 | -0.03 | 0.10 |
| Negative Emotion | 1.67 | 0.99 | 1.68 | 1.01 | -1.82 | .07 | -0.06 | -0.13 | 0.00 |
| Sadness | 0.33 | 0.32 | 0.34 | 0.33 | -0.80 | .43 | -0.03 | -0.10 | 0.04 |
| Anxiety | 0.45 | 0.40 | 0.45 | 0.42 | -1.79 | .07 | -0.06 | -0.13 | 0.01 |
| Cognitive Processes | 12.25 | 2.55 | 12.26 | 2.60 | -0.78 | .44 | -0.03 | -0.09 | 0.04 |
| Analytical Thinking | 57.88 | 17.66 | 57.83 | 17.82 | 0.34 | .73 | 0.01 | -0.06 | 0.08 |

*Note. N* = 835.

**Table S2**

*One-Way Between-Group Comparisons of All LIWC Outcomes*

|  | Test Statistic | |  |  | Effect Size | | | | | |
| --- | --- | --- | --- | --- | --- | --- | --- | --- | --- | --- |
|  | Type | Value | *p* | *p*_adj_ | Type | | Value | | Interpretation | |
| WC | χ^2^ | 406.927 | < .001 | < .001 | ε^2^_ordinal_ | .138 | | Medium | |  |
| Analytic | *F* | 18.442 | < .001 | < .001 | ε^2^_ordinal_ | .098 | | Medium | |  |
| Clout | χ^2^ | 374.209 | < .001 | < .001 | ε^2^_ordinal_ | .127 | | Medium | |  |
| Authentic | χ^2^ | 27.488 | < .001 | .004 | ε^2^_ordinal_ | .009 | | Very small | |  |
| Tone | χ^2^ | 396.946 | < .001 | < .001 | ε^2^_ordinal_ | .135 | | Medium | |  |
| WPS | χ^2^ | 13.107 | .022 | > .999 | ε^2^_ordinal_ | .004 | | Very small | |  |
| Sixltr | *F* _(trimmed-means)_ | 28.394 | < .001 | < .001 | ξ | .260 | | Small | |  |
| Dic | *F* _(trimmed-means)_ | 55.231 | < .001 | < .001 | ξ | .360 | | Medium | |  |
| function | *F* _(trimmed-means)_ | 9.462 | < .001 | < .001 | ξ | .243 | | Small | |  |
| pronoun | *F* _(trimmed-means)_ | 8.256 | < .001 | < .001 | ξ | .182 | | Small | |  |
| ppron | *F* | 13.170 | < .001 | < .001 | η^2^ | .073 | | Medium | |  |
| i | *F* _(trimmed-means)_ | 14.083 | < .001 | < .001 | ξ | .236 | | Small | |  |
| we | χ^2^ | 483.126 | < .001 | < .001 | ε^2^_ordinal_ | .164 | | Large | |  |
| you | χ^2^ | 72.899 | < .001 | < .001 | ε^2^_ordinal_ | .025 | | Small | |  |
| shehe | χ^2^ | 177.589 | < .001 | < .001 | ε^2^_ordinal_ | .060 | | Medium | |  |
| they | χ^2^ | 200.856 | < .001 | < .001 | ε^2^_ordinal_ | .068 | | Medium | |  |
| ipron | *F* _(trimmed-means)_ | 13.969 | < .001 | < .001 | ξ | .182 | | Small | |  |
| article | *F* | 29.808 | < .001 | < .001 | η^2^ | .151 | | Large | |  |
| prep | *F* _(trimmed-means)_ | 14.164 | < .001 | < .001 | ξ | .204 | | Small | |  |
| auxverb | *F* _(trimmed-means)_ | 8.477 | < .001 | < .001 | ξ | .155 | | Small | |  |
| adverb | *F* _(trimmed-means)_ | 20.469 | < .001 | < .001 | ξ | .214 | | Small | |  |
| conj | *F* _(trimmed-means)_ | 7.502 | < .001 | < .001 | ξ | .167 | | Small | |  |
| negate | *F* _(trimmed-means)_ | 25.302 | < .001 | < .001 | ξ | .333 | | Small | |  |
| verb | *F* | 14.601 | < .001 | < .001 | η^2^ | .079 | | Medium | |  |
| adj | *F* _(trimmed-means)_ | 17.000 | < .001 | < .001 | ξ | .209 | | Small | |  |
| compare | *F* _(trimmed-means)_ | 19.244 | < .001 | < .001 | ξ | .239 | | Small | |  |
| interrog | *F* _(trimmed-means)_ | 12.182 | < .001 | < .001 | ξ | .244 | | Small | |  |
| number | χ^2^ | 159.372 | < .001 | < .001 | ε^2^_ordinal_ | .054 | | Small | |  |
| quant | *F* _(trimmed-means)_ | 6.228 | < .001 | .001 | ξ | .148 | | Very small | |  |
| affect | *F* _(trimmed-means)_ | 75.664 | < .001 | < .001 | ξ | .449 | | Medium | |  |
| posemo | *F* _(trimmed-means)_ | 88.756 | < .001 | < .001 | ξ | .496 | | Medium | |  |
| negemo | χ^2^ | 284.916 | < .001 | < .001 | ε^2^_ordinal_ | .097 | | Medium | |  |
| anx | χ^2^ | 21.701 | < .001 | .057 | ε^2^_ordinal_ | .007 | | Very small | |  |
| anger | χ^2^ | 75.073 | < .001 | < .001 | ε^2^_ordinal_ | .025 | | Small | |  |
| sad | χ^2^ | 79.441 | < .001 | < .001 | ε^2^_ordinal_ | .027 | | Small | |  |
| social | *F* _(trimmed-means)_ | 119.964 | < .001 | < .001 | ξ | .537 | | Large | |  |
| family | χ^2^ | 77.720 | < .001 | < .001 | ε^2^_ordinal_ | .026 | | Small | |  |
| friend | χ^2^ | 347.487 | < .001 | < .001 | ε^2^_ordinal_ | .118 | | Medium | |  |
| female | χ^2^ | 77.026 | < .001 | < .001 | ε^2^_ordinal_ | .026 | | Small | |  |
| male | χ^2^ | 185.045 | < .001 | < .001 | ε^2^_ordinal_ | .063 | | Medium | |  |
| cogproc | *F* | 13.321 | < .001 | < .001 | η^2^ | .073 | | Medium | |  |
| insight | *F* _(trimmed-means)_ | 5.866 | < .001 | .003 | ξ | .164 | | Small | |  |
| cause | *F* _(trimmed-means)_ | 9.889 | < .001 | < .001 | ξ | .294 | | Small | |  |
| discrep | *F* _(trimmed-means)_ | 3.293 | .006 | .587 | ξ | .141 | | Very small | |  |
| tentat | *F* _(trimmed-means)_ | 9.676 | < .001 | < .001 | ξ | .235 | | Small | |  |
| certain | χ^2^ | 86.750 | < .001 | < .001 | ε^2^_ordinal_ | .029 | | Small | |  |
| differ | *F* _(trimmed-means)_ | 12.510 | < .001 | < .001 | ξ | .238 | | Small | |  |
| percept | *F* _(trimmed-means)_ | 34.996 | < .001 | < .001 | ξ | .427 | | Medium | |  |
| see | χ^2^ | 534.272 | < .001 | < .001 | ε^2^_ordinal_ | .181 | | Large | |  |
| hear | χ^2^ | 152.659 | < .001 | < .001 | ε^2^_ordinal_ | .052 | | Small | |  |
| feel | χ^2^ | 248.524 | < .001 | < .001 | ε^2^_ordinal_ | .084 | | Medium | |  |
| bio | χ^2^ | 577.560 | < .001 | < .001 | ε^2^_ordinal_ | .196 | | Large | |  |
| body | χ^2^ | 144.243 | < .001 | < .001 | ε^2^_ordinal_ | .049 | | Small | |  |
| health | χ^2^ | 716.275 | < .001 | < .001 | ε^2^_ordinal_ | .243 | | Large | |  |
| sexual | χ^2^ | 32.601 | < .001 | < .001 | ε^2^_ordinal_ | .011 | | Small | |  |
| ingest | χ^2^ | 295.953 | < .001 | < .001 | ε^2^_ordinal_ | .100 | | Medium | |  |
| drives | *F* _(trimmed-means)_ | 131.973 | < .001 | < .001 | ξ | .531 | | Large | |  |
| affiliation | χ^2^ | 618.027 | < .001 | < .001 | ε^2^_ordinal_ | .210 | | Large | |  |
| achieve | χ^2^ | 48.033 | < .001 | < .001 | ε^2^_ordinal_ | .016 | | Small | |  |
| power | *F* _(trimmed-means)_ | 1.538 | .176 | > .999 | ξ | .110 | | Very small | |  |
| reward | χ^2^ | 511.146 | < .001 | < .001 | ε^2^_ordinal_ | .174 | | Large | |  |
| risk | χ^2^ | 79.305 | < .001 | < .001 | ε^2^_ordinal_ | .027 | | Small | |  |
| focuspast | *F* _(trimmed-means)_ | 19.588 | < .001 | < .001 | ξ | .265 | | Small | |  |
| focuspresent | *F* _(trimmed-means)_ | 13.001 | < .001 | < .001 | ξ | .257 | | Small | |  |
| focusfuture | χ^2^ | 8.159 | .148 | > .999 | ε^2^_ordinal_ | .003 | | Very small | |  |
| relativ | *F* _(trimmed-means)_ | 16.125 | < .001 | < .001 | ξ | .226 | | Small | |  |
| motion | *F* _(trimmed-means)_ | 52.228 | < .001 | < .001 | ξ | .412 | | Medium | |  |
| space | *F* | 87.590 | < .001 | < .001 | η^2^ | .341 | | Large | |  |
| time | *F* _(trimmed-means)_ | 34.969 | < .001 | < .001 | ξ | .414 | | Medium | |  |
| work | χ^2^ | 109.664 | < .001 | < .001 | ε^2^_ordinal_ | .037 | | Small | |  |
| leisure | χ^2^ | 293.419 | < .001 | < .001 | ε^2^_ordinal_ | .100 | | Medium | |  |
| home | χ^2^ | 151.847 | < .001 | < .001 | ε^2^_ordinal_ | .052 | | Small | |  |
| money | χ^2^ | 63.708 | < .001 | < .001 | ε^2^_ordinal_ | .022 | | Small | |  |
| relig | χ^2^ | 207.303 | < .001 | < .001 | ε^2^_ordinal_ | .070 | | Medium | |  |
| death | χ^2^ | 72.680 | < .001 | < .001 | ε^2^_ordinal_ | .025 | | Small | |  |
| informal | χ^2^ | 115.040 | < .001 | < .001 | ε^2^_ordinal_ | .039 | | Small | |  |
| swear | χ^2^ | 97.443 | < .001 | < .001 | ε^2^_ordinal_ | .033 | | Small | |  |
| netspeak | χ^2^ | 189.191 | < .001 | < .001 | ε^2^_ordinal_ | .064 | | Medium | |  |
| assent | χ^2^ | 164.938 | < .001 | < .001 | ε^2^_ordinal_ | .056 | | Small | |  |
| nonflu | χ^2^ | 26.293 | < .001 | .007 | ε^2^_ordinal_ | .009 | | Very small | |  |
| filler | χ^2^ | 43.734 | < .001 | < .001 | ε^2^_ordinal_ | .015 | | Small | |  |
| AllPunc | *F* _(trimmed-means)_ | 2.308 | .043 | > .999 | ξ | .119 | | Very small | |  |
| Period | *F* _(trimmed-means)_ | 2.153 | .058 | > .999 | ξ | .114 | | Very small | |  |
| Comma | *F* | 1.045 | .390 | > .999 | η^2^ | .006 | | Very small | |  |
| Colon | χ^2^ | 28.315 | < .001 | .003 | ε^2^_ordinal_ | .010 | | Very small | |  |
| SemiC | χ^2^ | 35.765 | < .001 | < .001 | ε^2^_ordinal_ | .012 | | Small | |  |
| QMark | χ^2^ | 109.854 | < .001 | < .001 | ε^2^_ordinal_ | .037 | | Small | |  |
| Exclam | χ^2^ | 51.270 | < .001 | < .001 | ε^2^_ordinal_ | .017 | | Small | |  |
| Dash | χ^2^ | 26.941 | < .001 | .006 | ε^2^_ordinal_ | .009 | | Very small | |  |
| Quote | χ^2^ | 87.304 | < .001 | < .001 | ε^2^_ordinal_ | .030 | | Small | |  |
| Apostro | χ^2^ | 15.918 | .007 | .673 | ε^2^_ordinal_ | .005 | | Very small | |  |
| Parenth | χ^2^ | 26.968 | < .001 | .005 | ε^2^_ordinal_ | .009 | | Very small | |  |
| OtherP | χ^2^ | 15.943 | .007 | .666 | ε^2^_ordinal_ | .005 | | Very small | |  |

**Figure S1**

Significant Differences Between Report Groups on Age

*
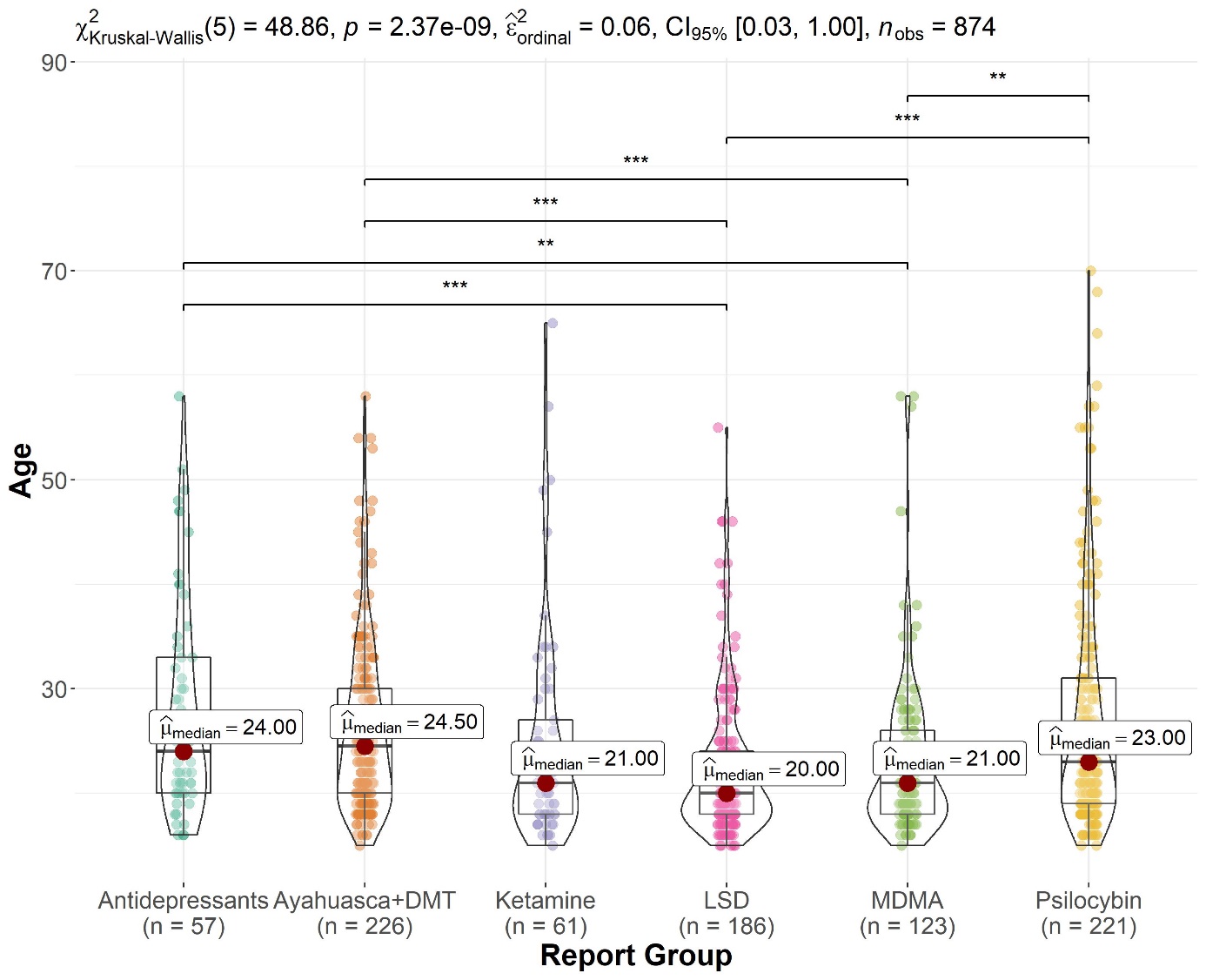
*

Note. ** p < .01, *** p < .001.

**Figure S2**

Significant Differences Between Report Groups on Body Weight


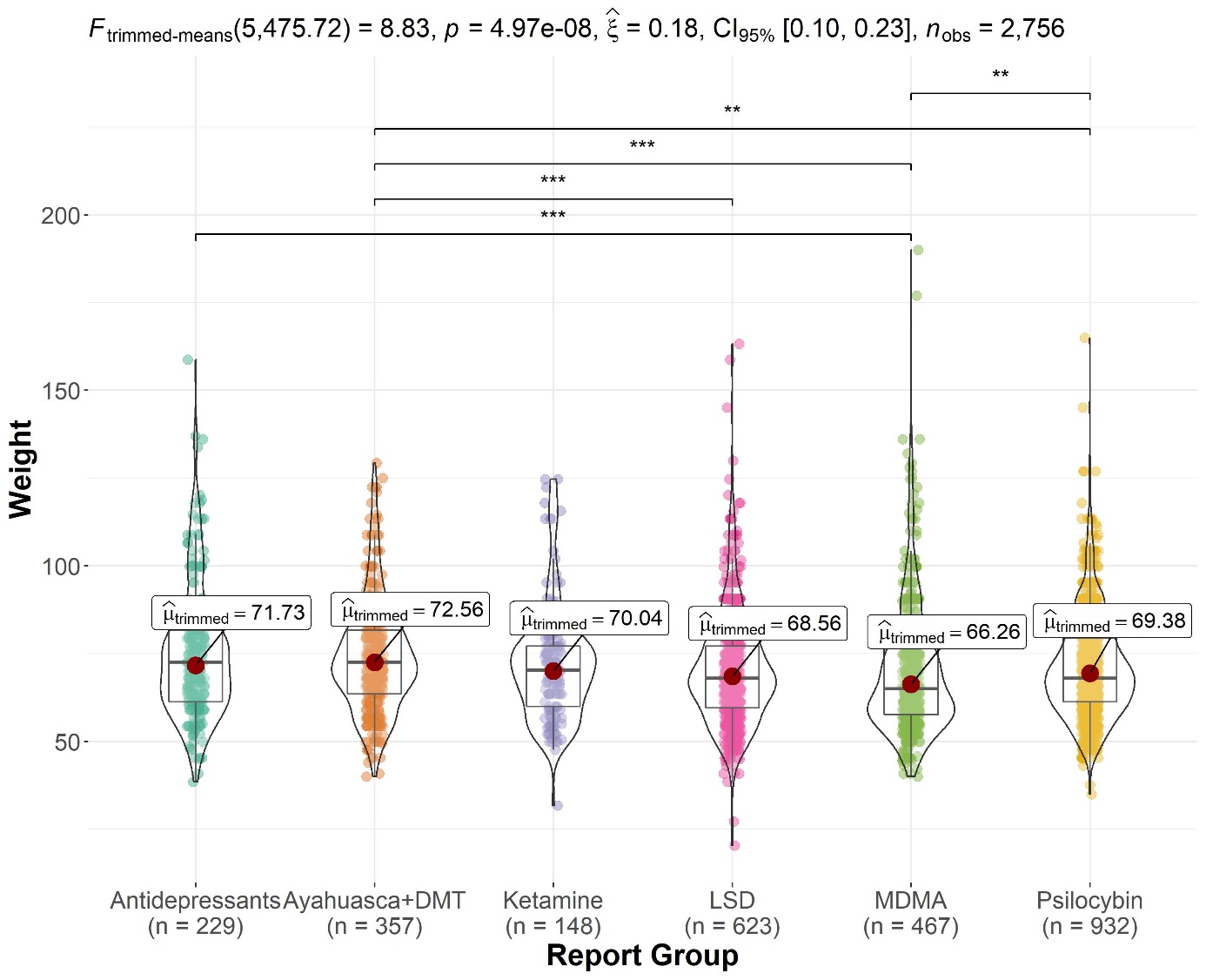


*Note*. ** *p* < .01, *** *p* < .001.

**Figure S3**

Significant Differences Between Report Groups on Proportion of Affective Process Words

*
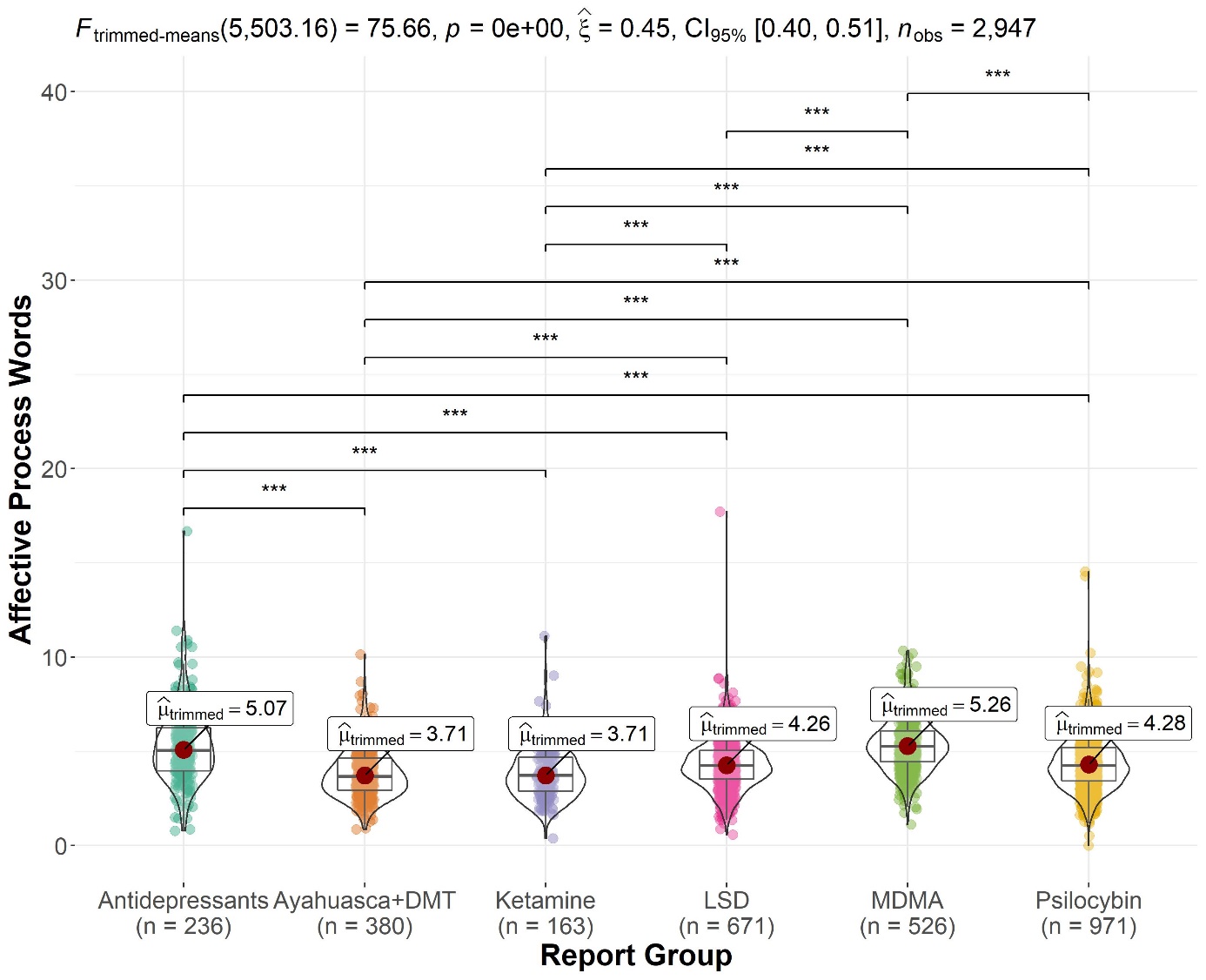
*

Note. *** p < .001.

**Figure S4**

Significant Differences Between Report Groups on Proportion of Sadness Words

*
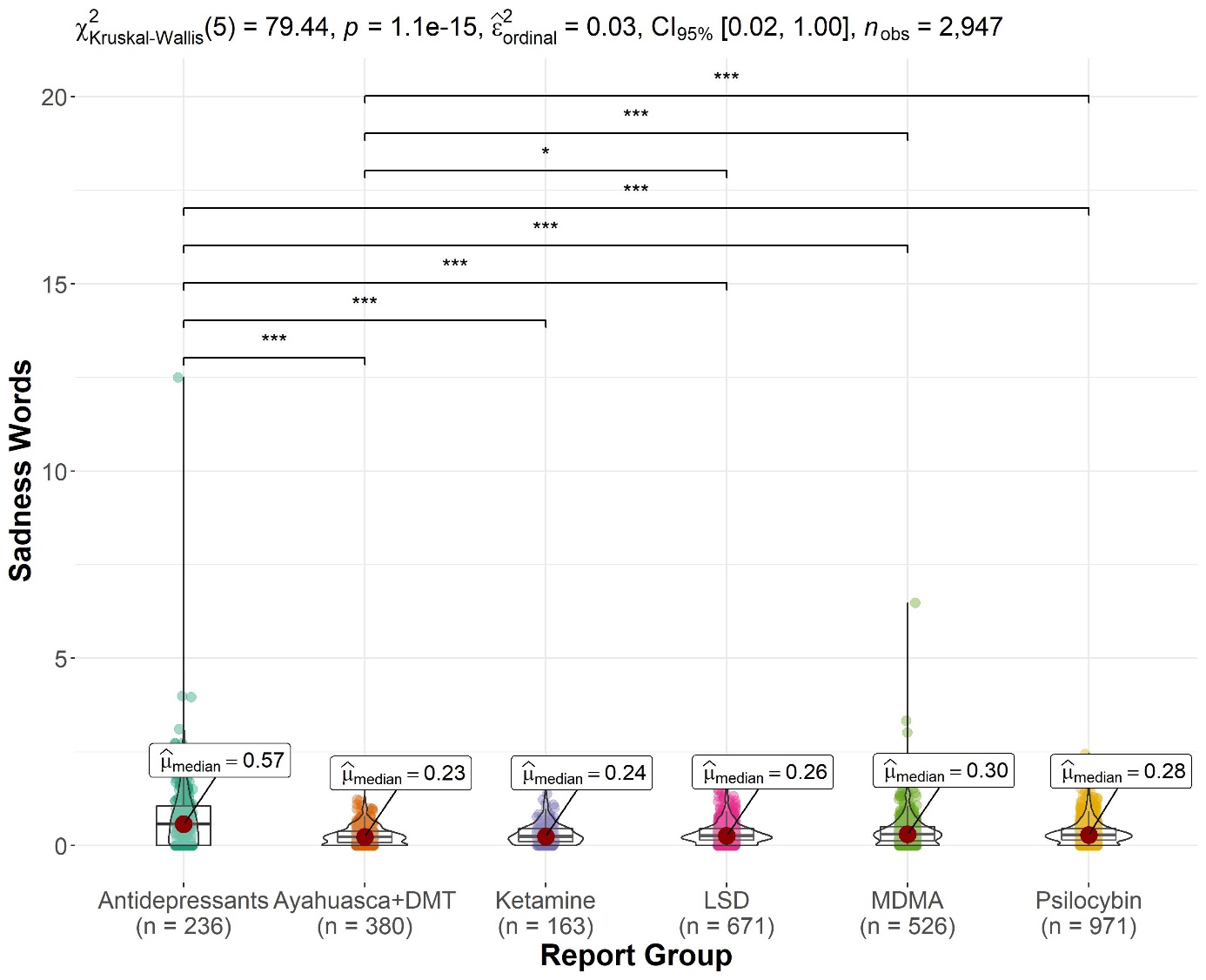
*

Note. * p < .05, *** p < .001.

**Figure S5**

Significant Differences Between Report Groups on Proportion of Anxiety Words

*
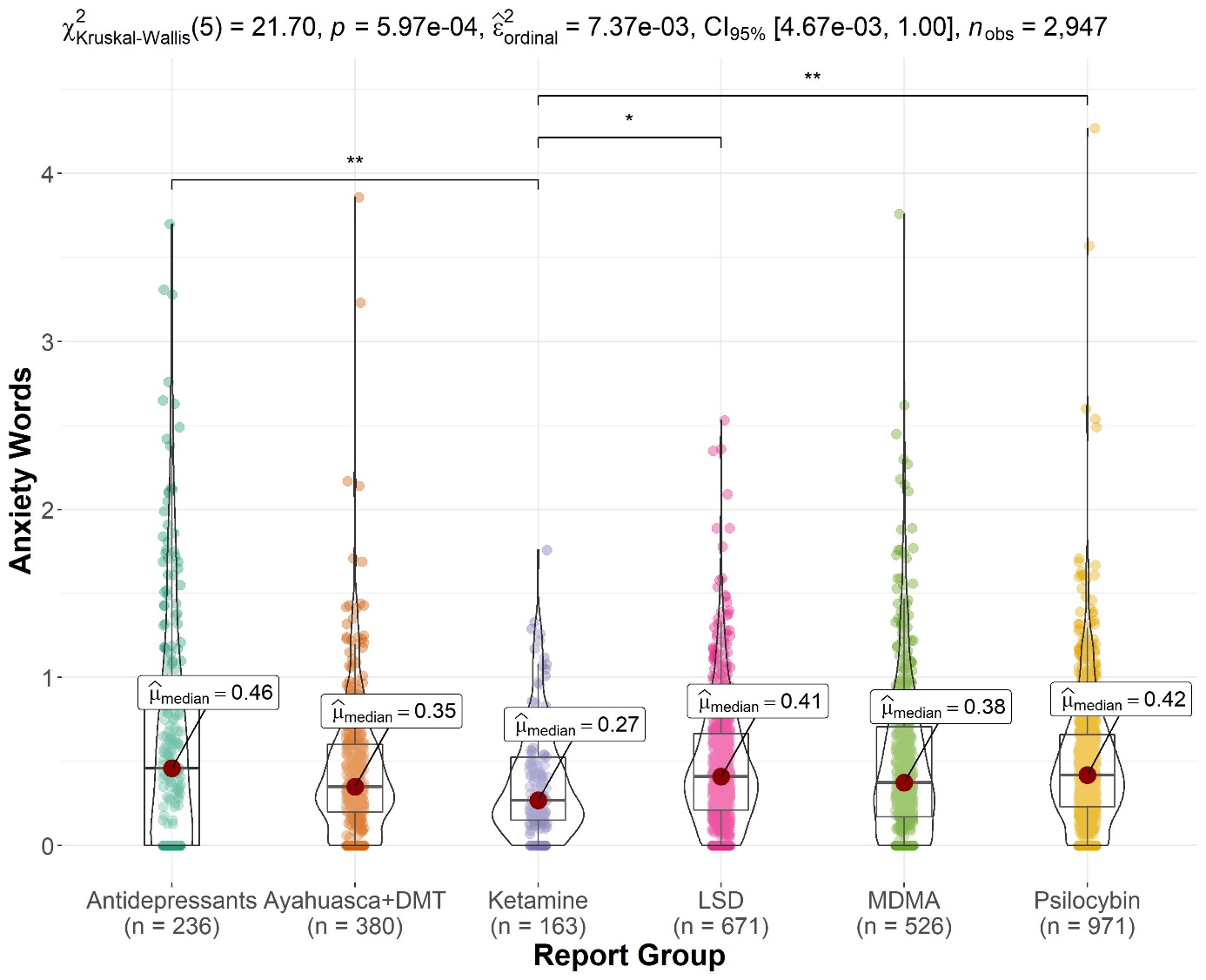
*

Note. * p < .05, ** p < .01.

**Figure S6**

Significant Differences Between Report Groups on Proportion of Negative Emotion Words


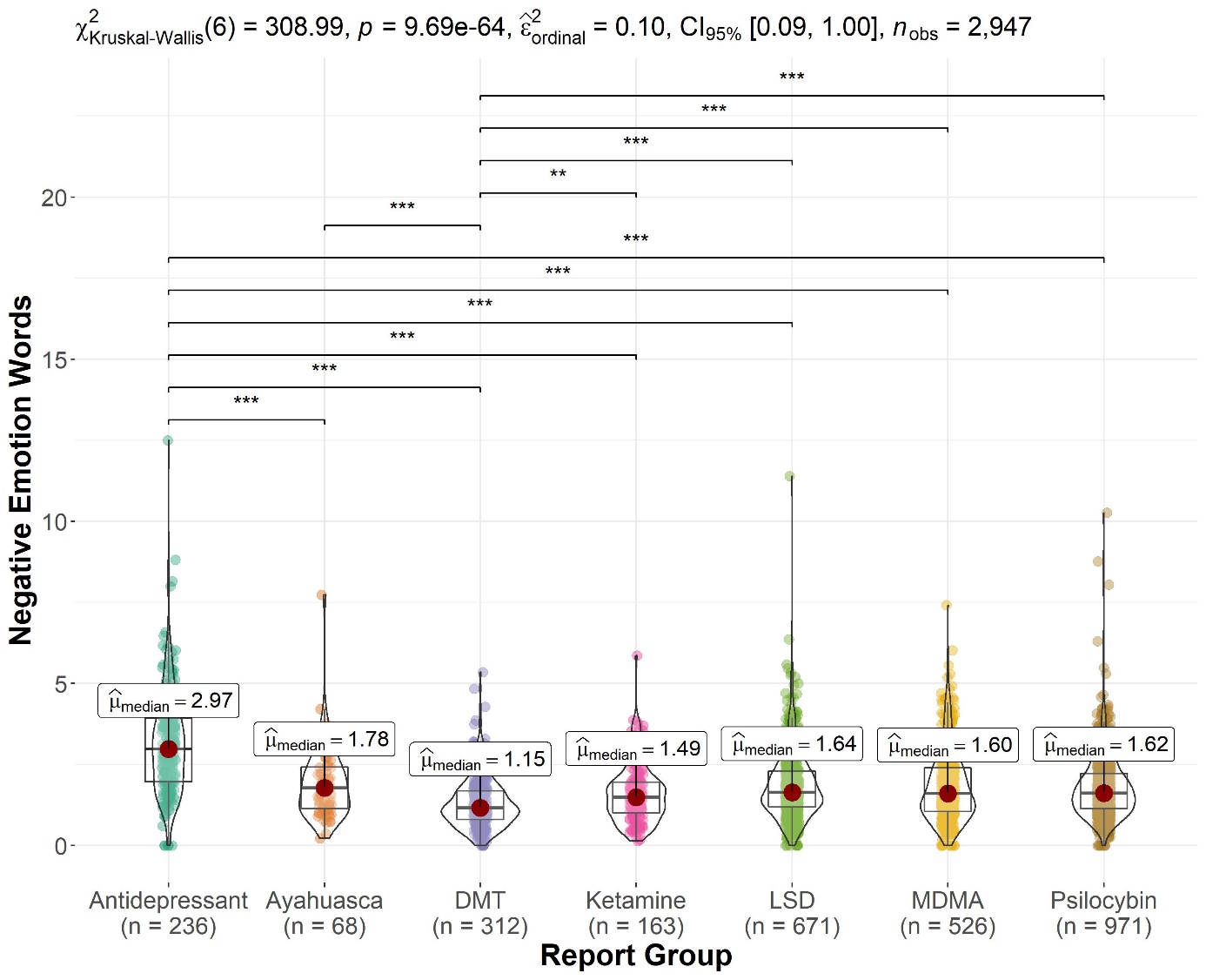


*Note*. ** *p* < .01, *** *p* < .001.

**Figure S7**

Significant Differences Between Report Groups on Hood’s M Scale Semantic Similarity Index


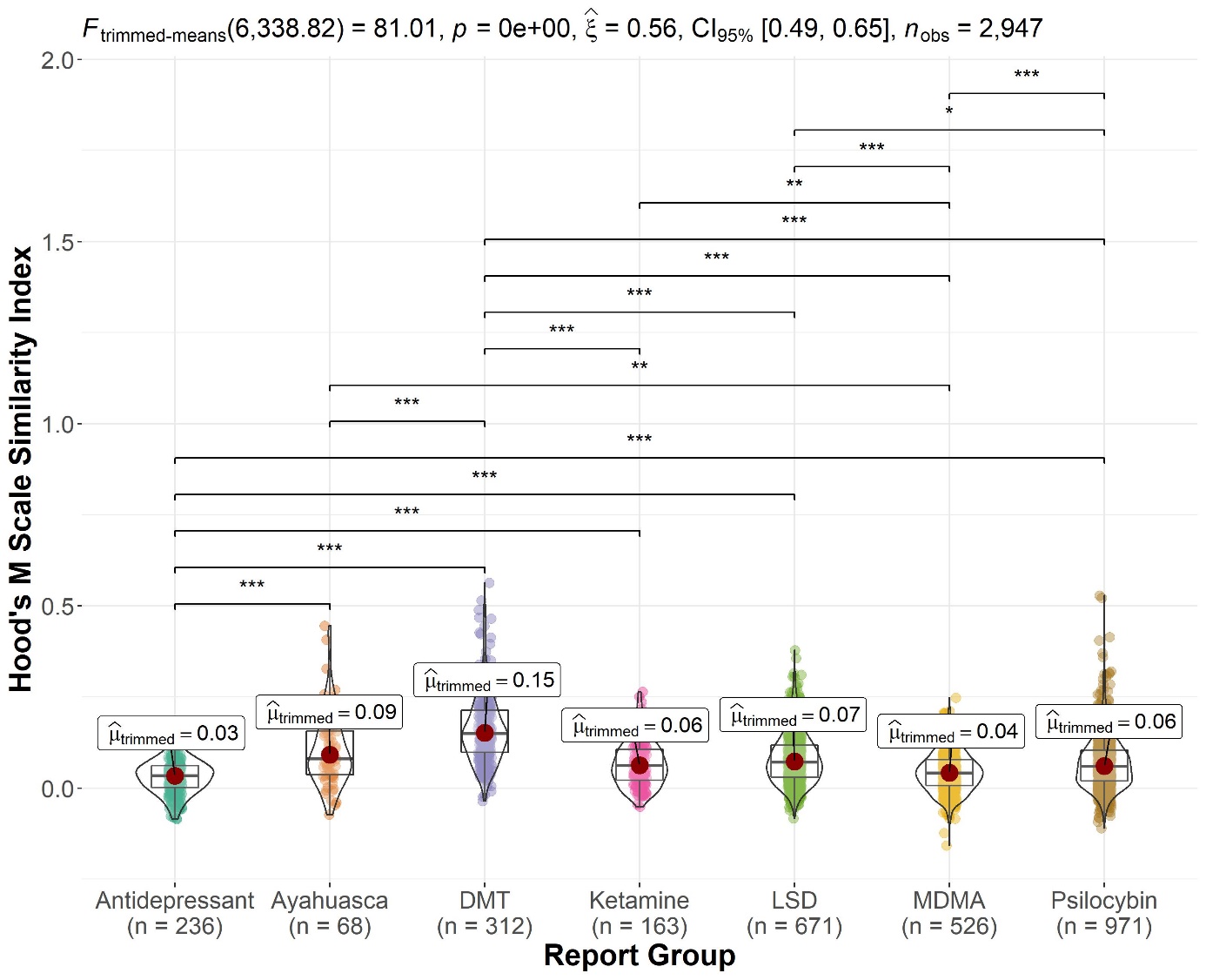


*Note*. * *p* < .05, ** *p* < .01, *** *p* < .001.

**Figure S8**

Significant Differences Between Report Groups on Proportion of First-Person Plural Words


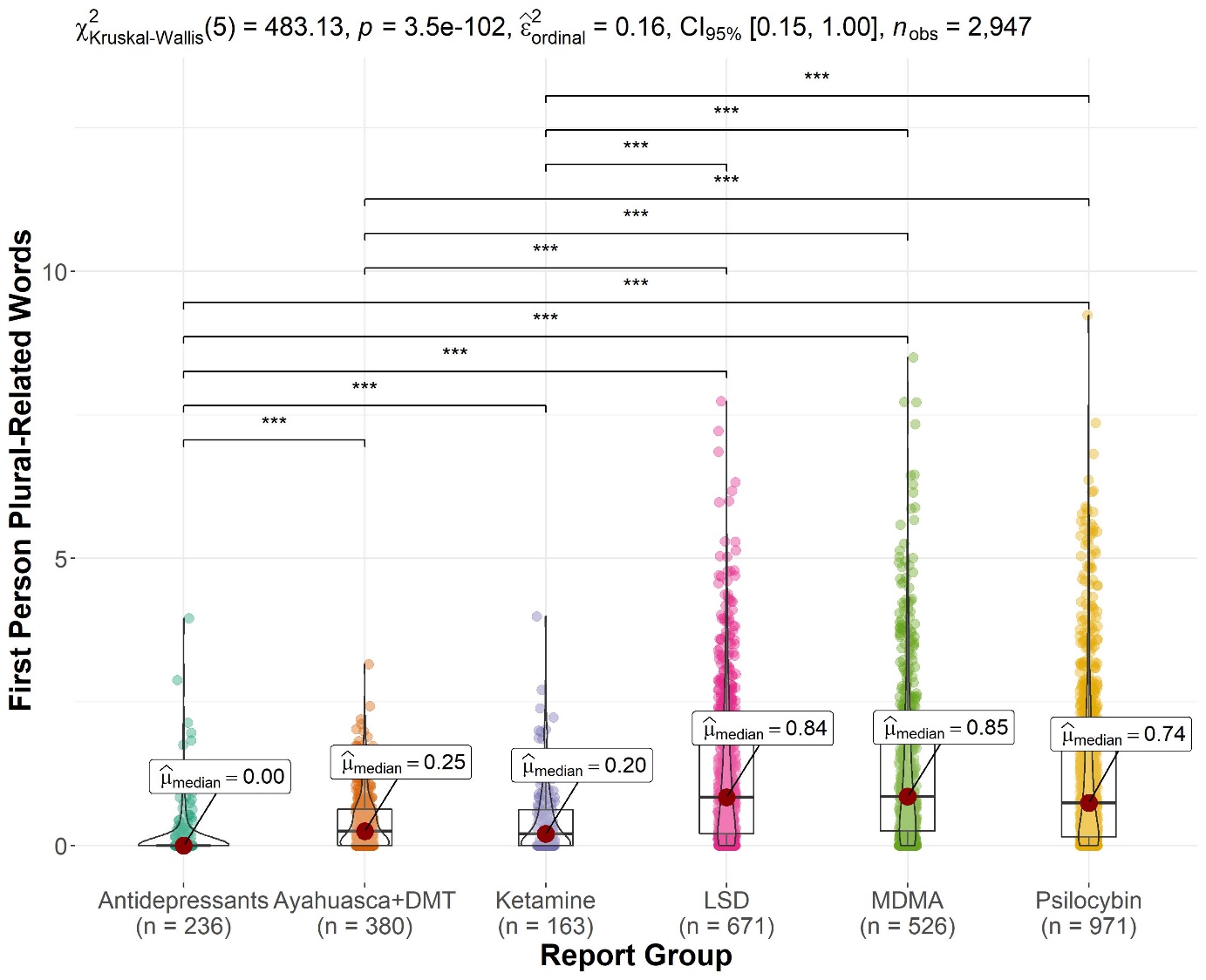


*Note.* *** *p* < .001.

**Figure S9**

Significant Differences Between Report Groups on Proportion of Articles


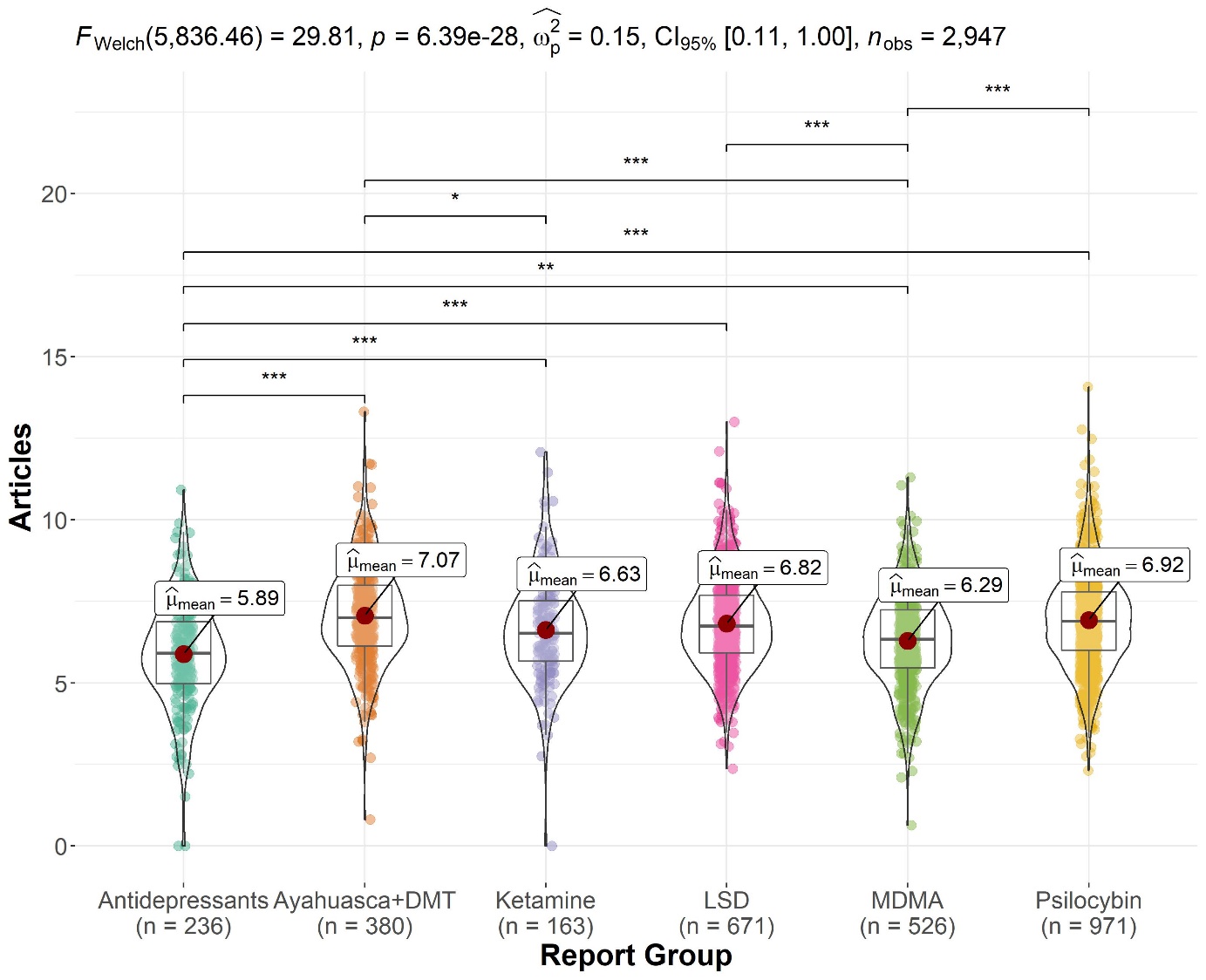


Note. * p < .05, ** p < .01, *** p < .001.

**Figure S10**

Significant Differences Between Report Groups on Social Process Word Proportions


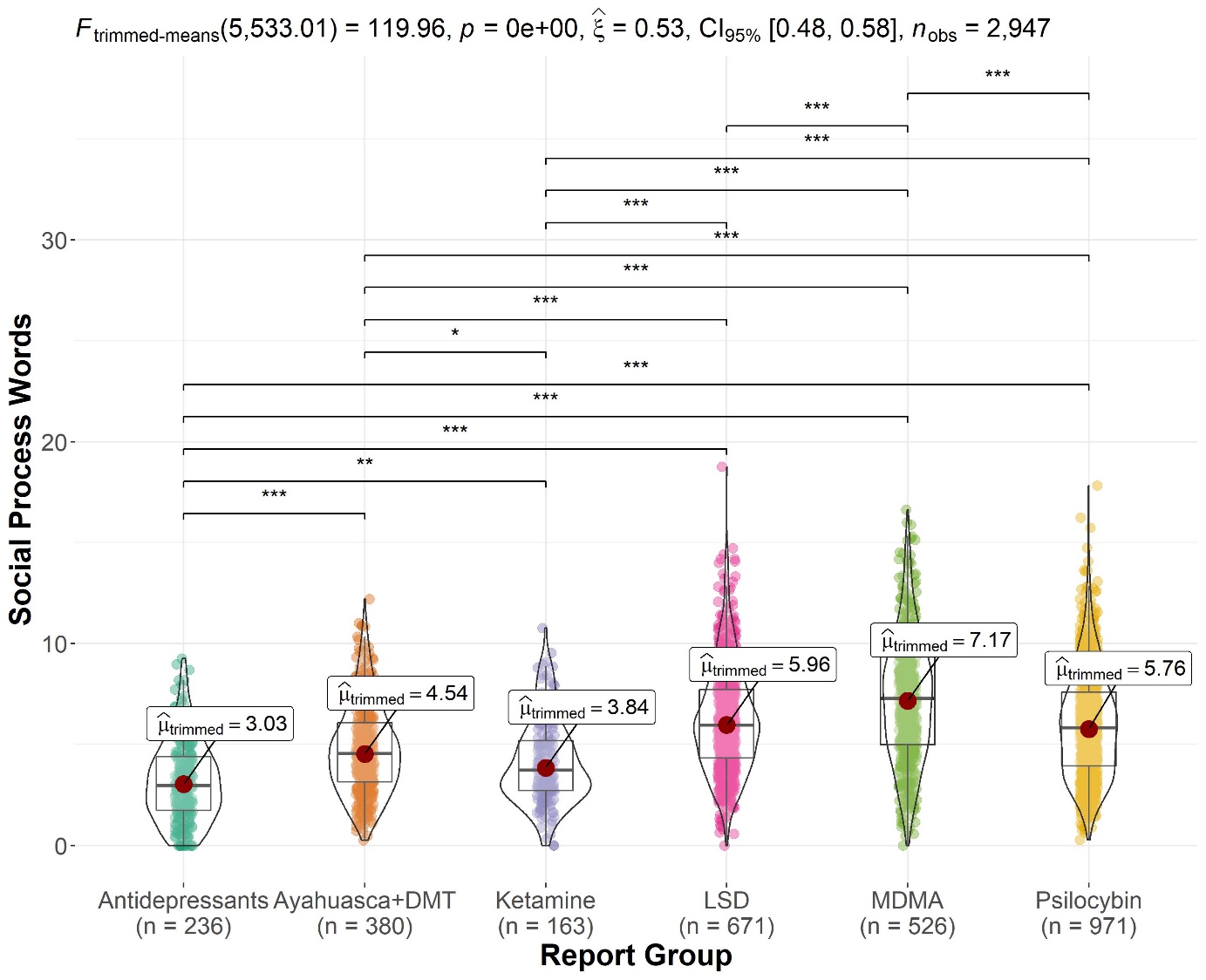


*Note*. * *p* < .05, ** *p* < .01, *** *p* < .001.

**Figure S11**

Significant Differences Between Report Groups on Vision-Related Word Proportions


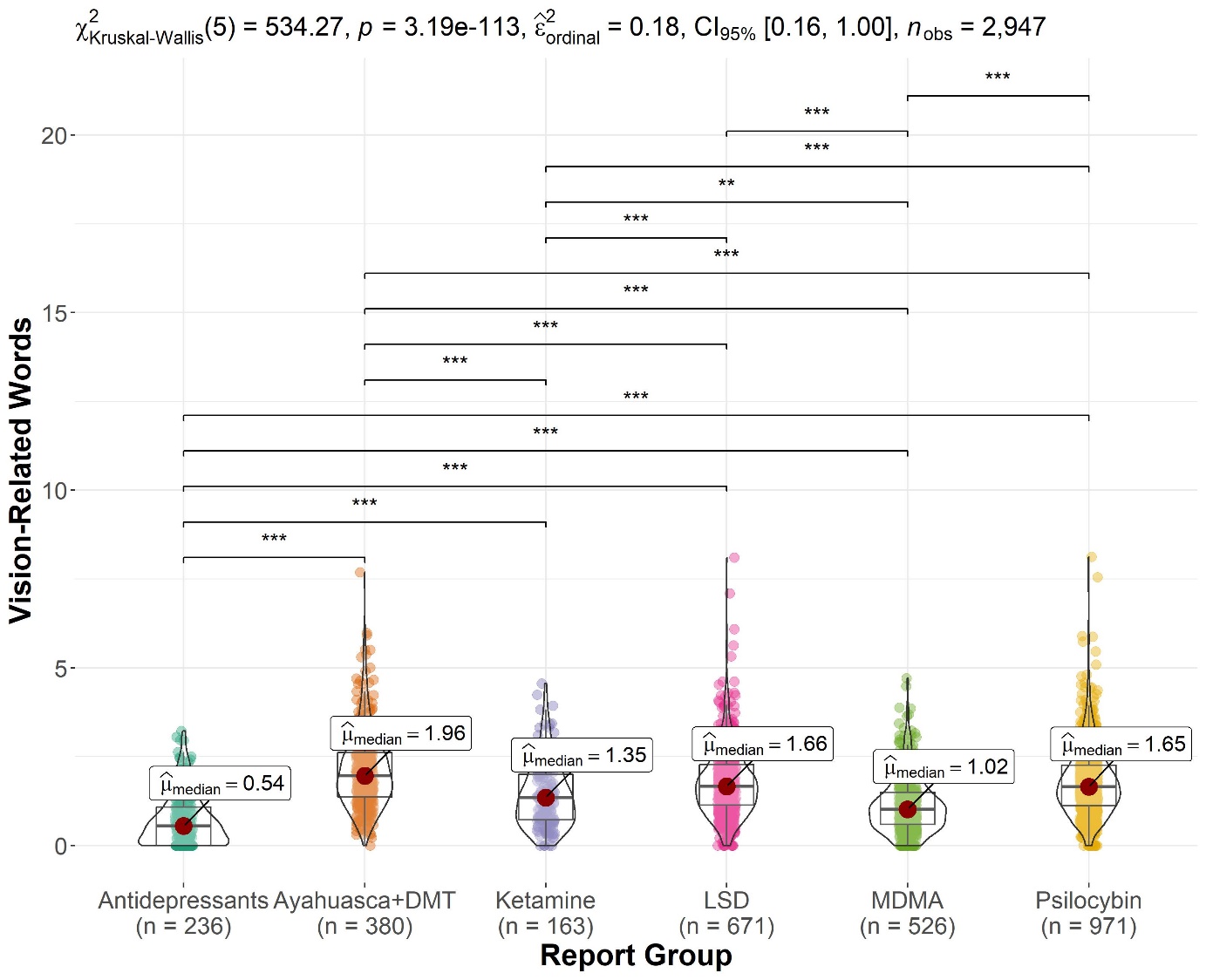


*Note*. ** *p* < .01, *** *p* < .001.

**Figure S12**

Significant Differences Between Report Groups on Biological Process Word Proportions


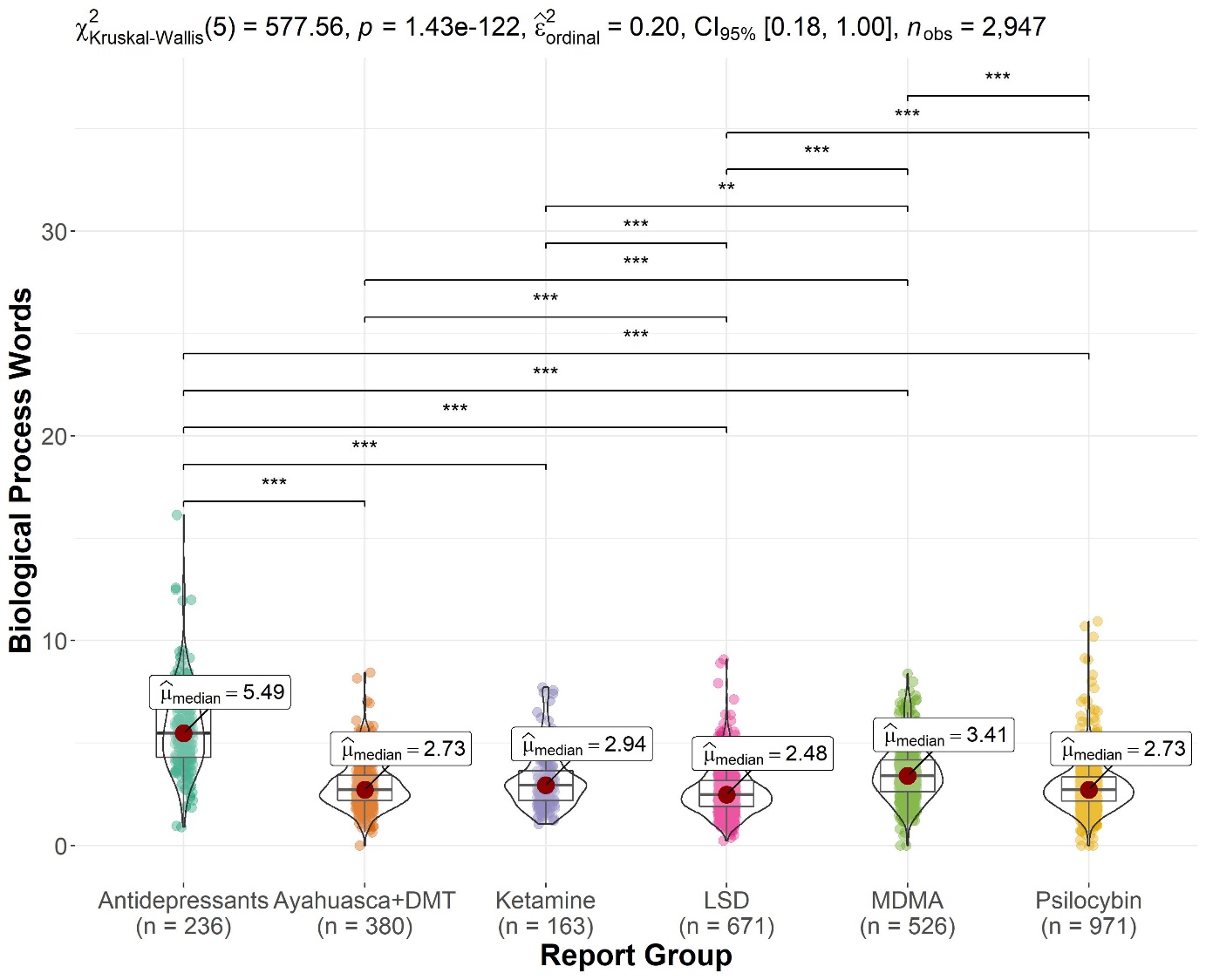


*Note*. ** *p* < .01, *** *p* < .001.

**Figure S13**

Significant Differences Between Report Groups on Health-Related Word Proportions


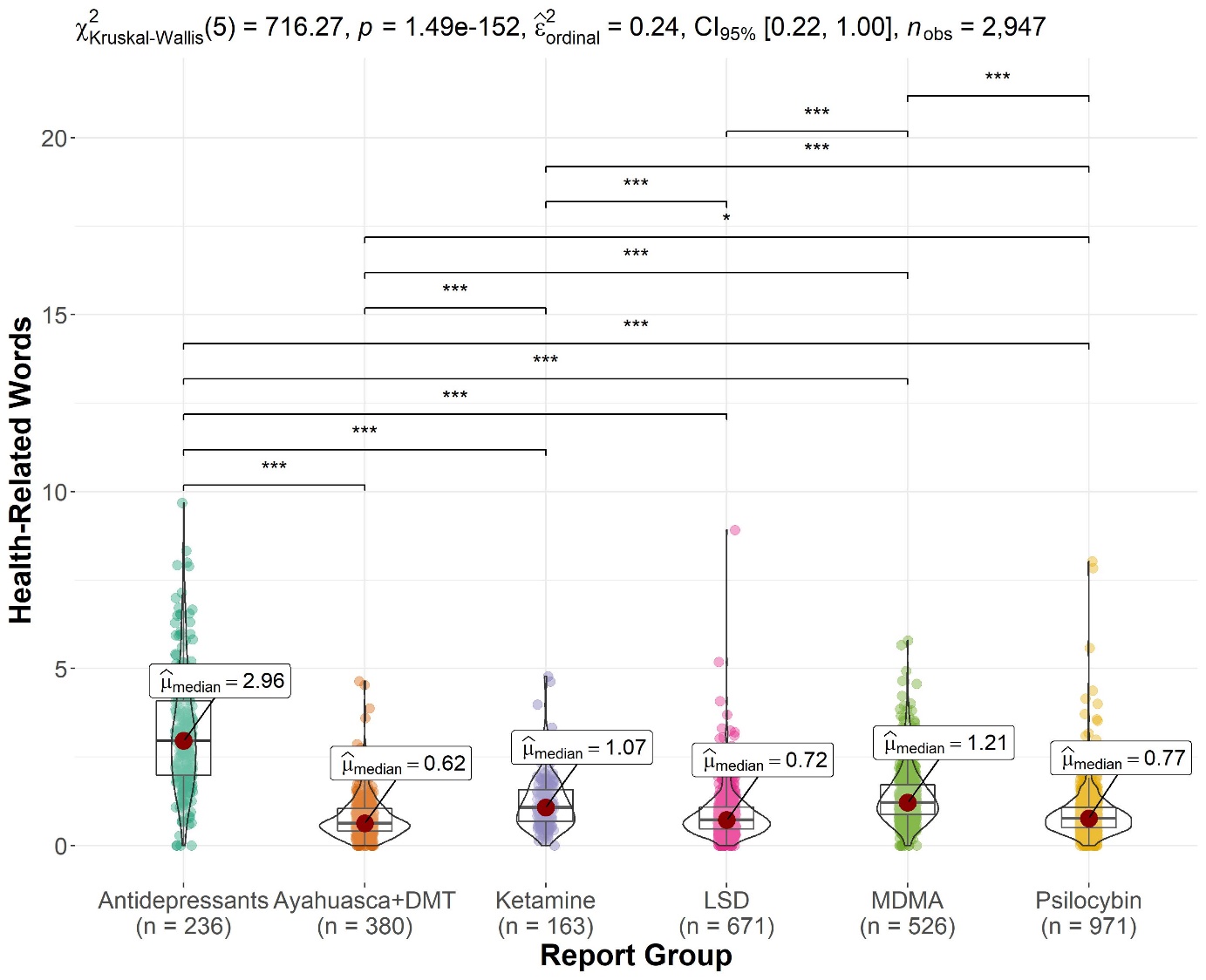


*Note*. * *p* < .05, *** *p* < .001.

**Figure S14**

Significant Differences Between Report Groups on Drive-Related Word Proportions


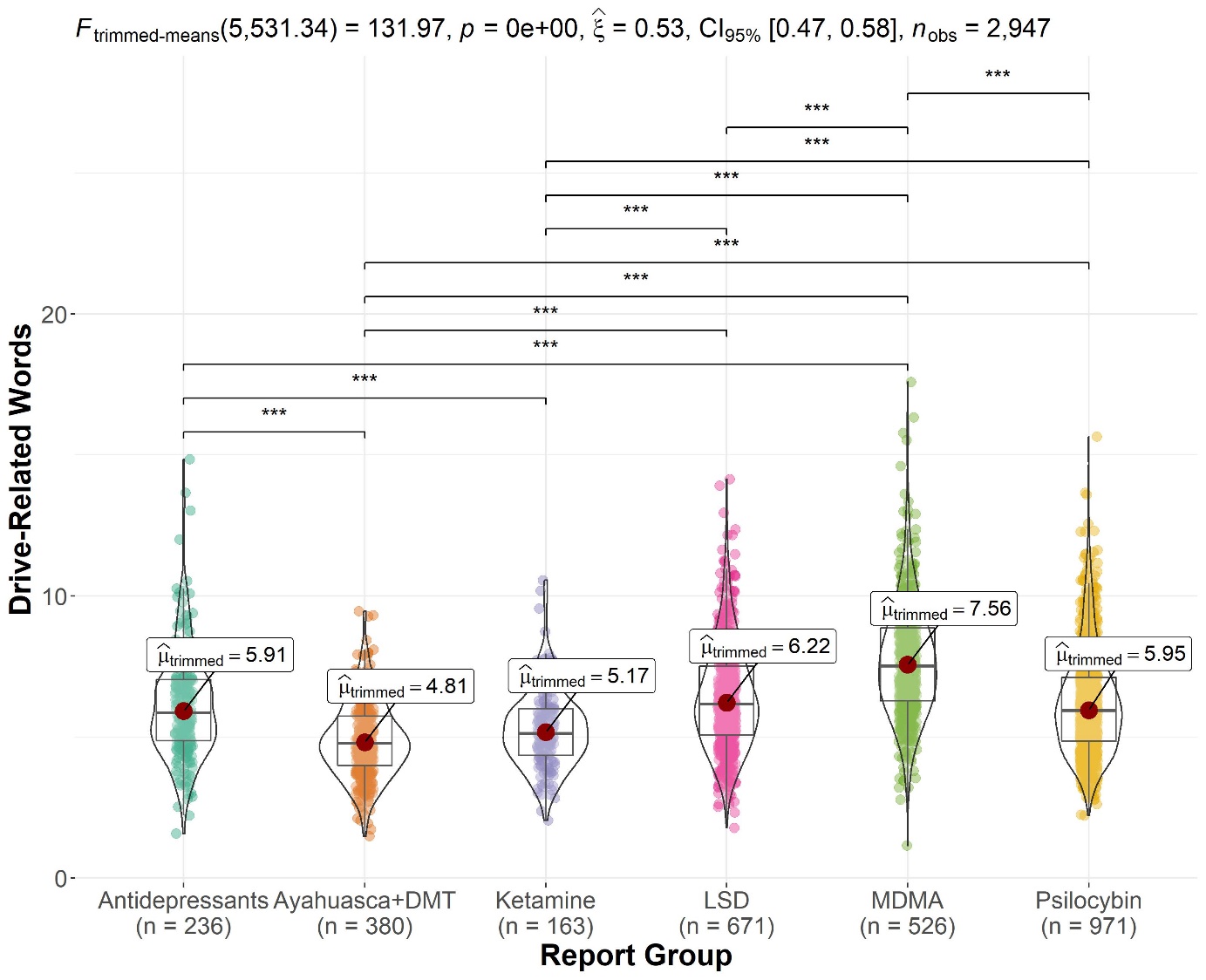


*Note*. *** *p* < .001.

**Figure S15**

Significant Differences Between Report Groups on Affiliation-Related Word Proportions


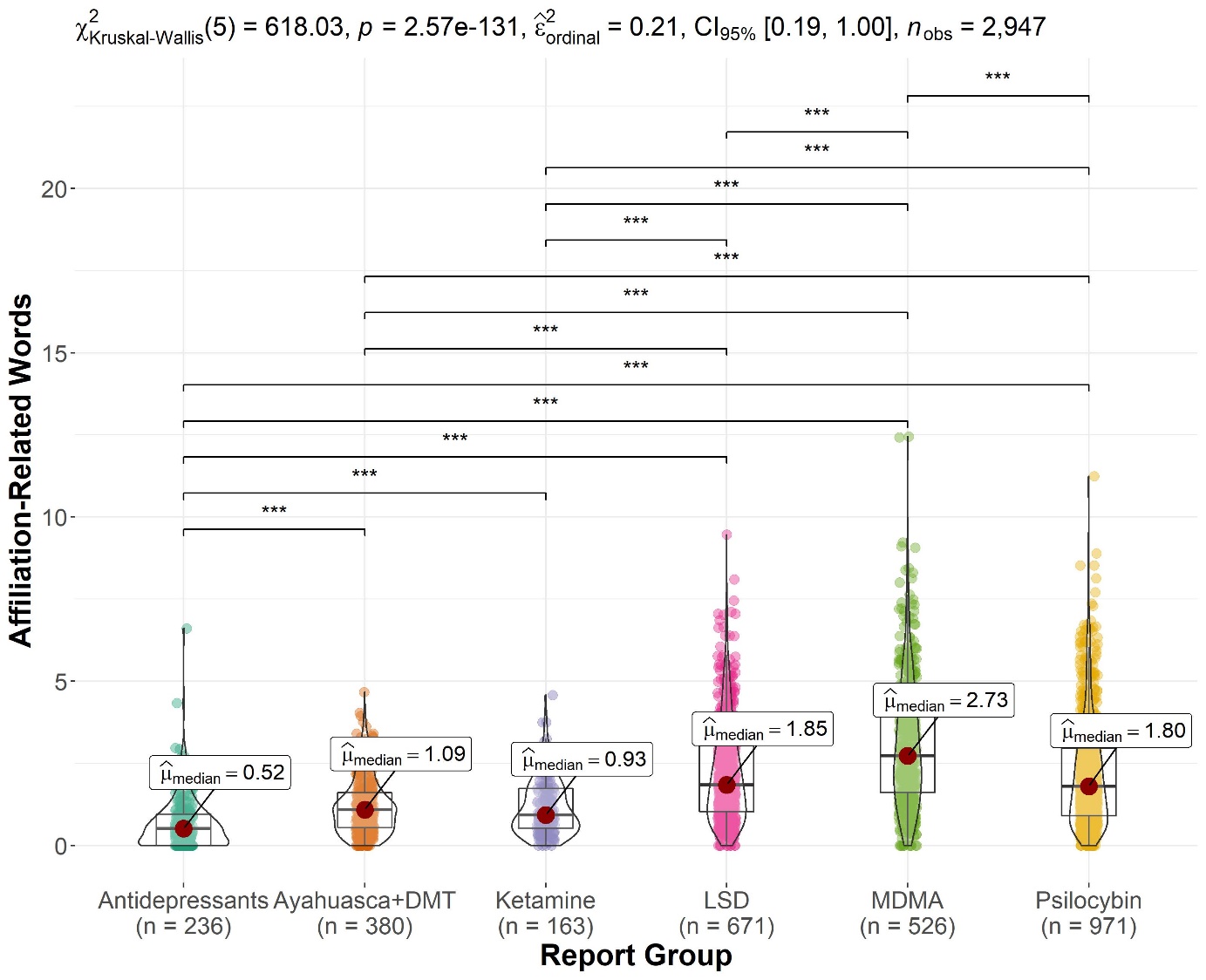


*Note*. *** *p* < .001.

**Figure S16**

Significant Differences Between Report Groups on Reward-Related Word Proportions


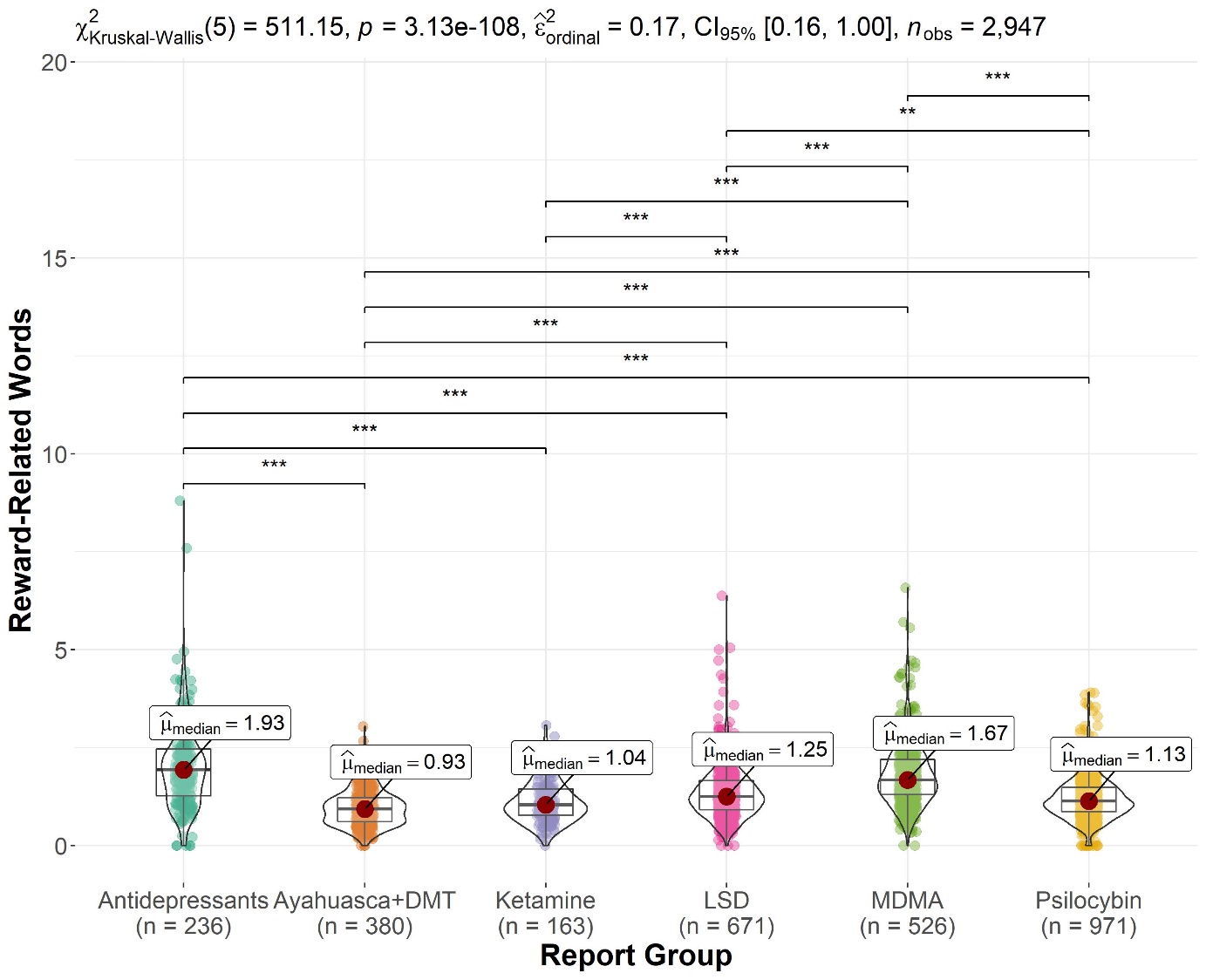


*Note*. ** *p* < .01, *** *p* < .001.

**Figure S17**

Significant Differences Between Report Groups on Space-Related Word Proportions


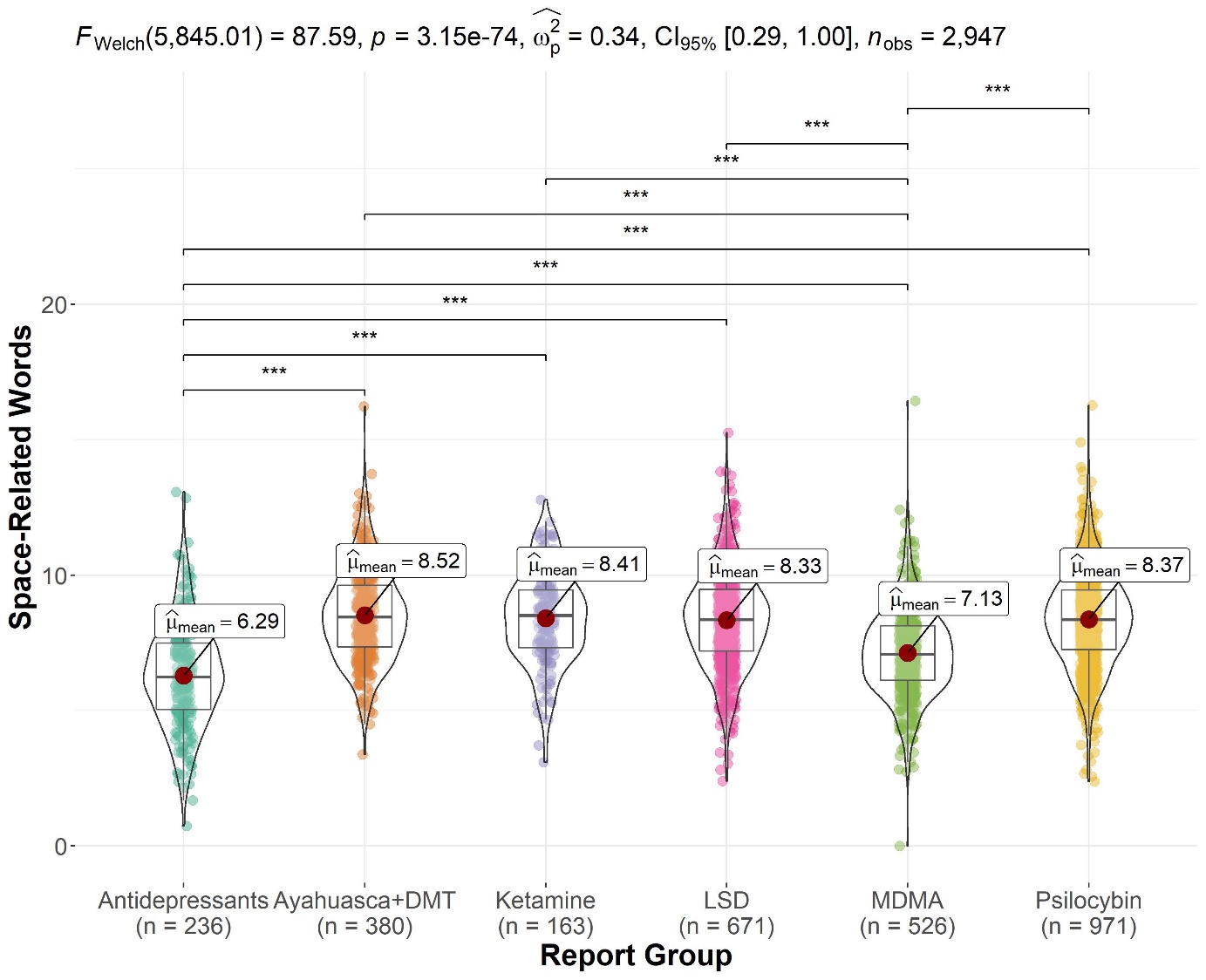


*Note*. *** *p* < .001.
